# Systematic Profiling of the Multicomponents and Authentication of Erzhi Pill by UHPLC/Q-Orbitrap-MS Oriented Rapid Polarity-Switching Data-Dependent Acquisition and Selective Monitoring of the Chemical Markers Deduced from Fingerprint Analysis

**DOI:** 10.3390/molecules23123143

**Published:** 2018-11-30

**Authors:** Li Jia, Lingling Fu, Xiaoyan Wang, Wenzhi Yang, Hongda Wang, Tiantian Zuo, Chunxia Zhang, Ying Hu, Xiumei Gao, Lifeng Han

**Affiliations:** 1 Tianjin State Key Laboratory of Modern Chinese Medicine, Tianjin University of Traditional Chinese Medicine, 312 Anshanxi Road, Tianjin 300193, China; jia10309@126.com (L.J.); nuncafuling1994@163.com (L.F.); 14741372177@163.com (X.W.); 17862987156@163.com (H.W.); 13553170361@163.com (T.Z.); 18202669277@163.com (C.Z.); huying3916@163.com (Y.H.); gaoxiumei@tjutcm.edu.cn (X.G.); 2 Tianjin Key Laboratory of TCM Chemistry and Analysis, Tianjin University of Traditional Chinese Medicine, 312 Anshanxi Road, Tianjin 300193, China

**Keywords:** Erzhi Pill, UHPLC/Q-Orbitrap-MS, polarity-switching data-dependent acquisition, identity marker, authentication, selective ion monitoring

## Abstract

The analytical platform UHPLC/Q-Orbitrap-MS offers a solution to quality investigation of TCM with high definiteness. Using Erzhi Pill (EZP) as a case, we developed UHPLC/Q-Orbitrap-MS based approaches to achieve systematic multicomponent identification and rapid authentication. Comprehensive multicomponent characterization of EZP was performed by negative/positive switching data-dependent high-energy collision-induced dissociation-MS^2^ (HCD-MS^2^) after 25 min chromatographic separation. By reference compounds comparison, elemental composition analysis, fragmentation pathways interpretation, and retrieval of an in-house library, 366 compounds were separated and detected from EZP, and 96 thereof were structurally characterized. The fingerprints of two component drugs (Ligustri Lucidi Fructus, LLF; Ecliptae Herba, EH) for EZP were analyzed under the same LC-MS condition by full scan in negative mode. In combination with currently available pharmacological reports, eight compounds were deduced as the ‘identity markers’ of EZP. Selective ion monitoring (SIM) of eight marker compounds was conducted to authenticate six batches of EZP samples. Both LLF and EH could be detected from all EZP samples by analyzing the SIM spectra, which could indicate their authenticity. Conclusively, UHPLC/Q-Orbitrap-MS by rapid polarity switching could greatly expand the potency of untargeted profiling with high efficiency, and SIM of multiple chemical markers rendered a practical approach enabling the authentication of TCM formulae.

## 1. Introduction

Investigations regarding the quality of traditional Chinese medicine (TCM) are commonly regarded as a systematic project [1]. Complexity in the chemical compositions of TCM (particularly the formulae), featuring co-existence of various primary and secondary botanical metabolites with sharply different contents, wide spans of molecular mass and polarity, and different acidity/basicity, raises great challenges for performing quality investigations of TCM [2,3,4,5,6,7,8]. Multicomponent characterization (to clarify what chemicals are involved), qualitative identification (to identify the source of raw materials or to authenticate the use of TCM materials in formulae), and content determination of assigned chemical markers (to quantify the content variations of some quality markers), are three key steps involved in quality investigation of TCM. The ongoing development of analytical technologies, especially LC-MS, offers some mature solutions to quality evaluation of TCM as well as TCM formulae [9].

Versatile MS scan methods and fragmentation mechanisms are currently available on high-resolution mass spectrometry (HRMS) in support of large-scale metabolites profiling and quantitation [10]. Comprehensive metabolites profiling strategies can be established in an untargeted mode by data-dependent (DDA) or data-independent acquisition (DIA) approaches. Comparatively, DIA by MS^E^ [11] or AIF (all ions fragmentation) [12] are more powerful in characterizing minor components than DDA, especially when chromatographic separation is insufficient, while the MS^n^ spectra obtained by DDA are easier to interpret than those recorded by DIA. On the other hand, different fragmentation mechanisms—Involving CID (collision-induced dissociation), HCD (high-energy collision-induced dissociation), and PQD (pulsed-Q dissociation) are alternatives on the hybrid linear ion-trap/orbitrap-MS (LTQ-Orbitrap-MS) platform. Differential and complementary fragmentation information can be obtained by integrated use of multiple fragmentation modes [13,14].

Erzhi Pill (EZP) is a reputable TCM formula showing liver-nourishing and kidney-enriching properties. EZP is prepared from Ligustri Lucidi Fructus (LLF, Ligustrum lucidum Ait.) and Ecliptae Herba (EH, Eclipta prostrata L.) in equal amounts. Diverse pharmaceutical effects have been reported for EZP, such as liver protection, immunoregulation, anti-hepatic fibrosis, anti-aging, anti-fatigue, anti-cancer, anti-diabetics, promoting coagulation, and improving memory, etc. [15]. *Chinese Pharmacopoeia* standard of EZP (2015 version) utilizes microscopic features and TLC for the authentication of LLF and EH, respectively, and specnuezhenide (C_31_H_42_O_17_) as the single quantitative marker for quality evaluation [16]. Actually, only by monitoring these very limited chemical markers, the authenticity and the holistic quality of TCM formulae are difficult to be assured. Simultaneous monitoring of multiple chemical markers has been proven to be powerful for identifying TCM formulae and even differentiating the contained congeneric TCM species [3,17]. Based on a recent UHPLC/QTOF-MS analysis report, up to six different subclasses of botanical metabolites (including iridoids, triterpenoids, phenylethanols, phenolic acids, flavonoids, and coumarins) have been qualitatively characterized from EZP [18]. However, in this work, only eight reference compounds were used.

**Figure 1:**
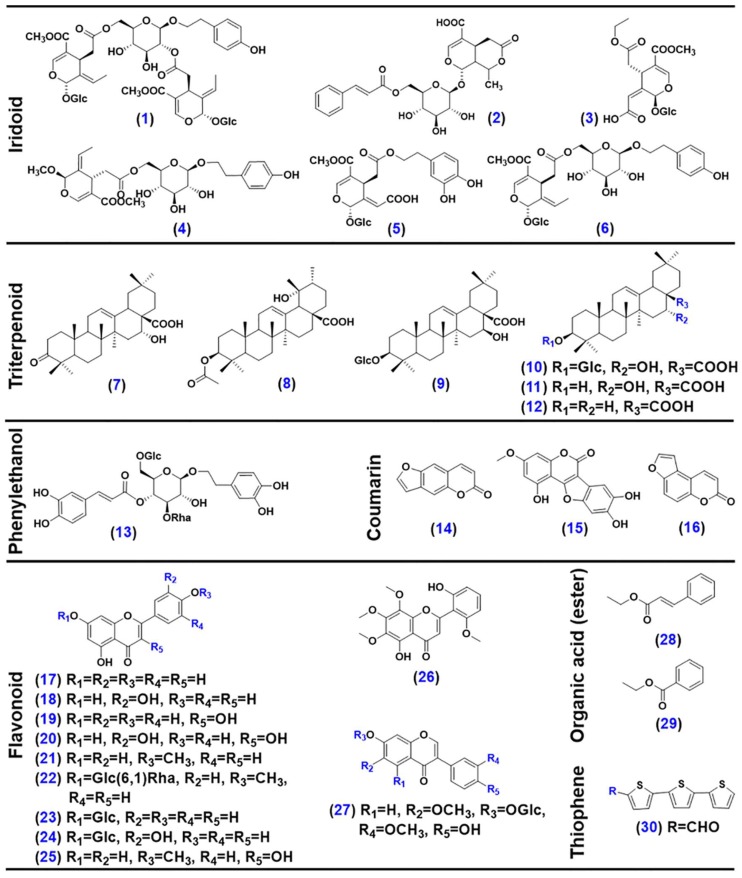
Chemical structures of 30 reference compounds, representative of seven different categories of plant secondary metabolites that were ever reported from Ligustri Lucidi Fructus and Ecliptae Herba.

The aim of this work was to establish UHPLC/Q-Orbitrap-MS approaches to facilitate the comprehensive, accurate metabolites profiling, and holistic, efficient authentication of EZP. Full MS/dd-MS^2^ (full scan/data-dependent MS^2^) by polarity switching (ESI−/ESI+) within one injection analysis was set to systematically characterize the multicomponents of EZP after UHPLC separation on a reversed-phase sub-2 µm particles packed BEH C18 column. The HCD-MS^2^ fragmentation behaviors of 30 reference compounds, representative of seven subclasses of natural compounds (Figure 1), was investigated to assist the structural elucidation of EZP compounds. ‘Identity markers’ of EZP were established by fingerprint analyses of two constituent TCM species (LLF and EH) under the same LC-MS condition as that used to analyze EZP. SIM (selective ion monitoring) was applied to the targeted monitoring of the ‘identity markers’ rapidly achieving the authentication of EZP. Hopefully, it can be an example for the comprehensive chemical profiling and authentication of TCM formulae by the UHPLC/Q-Orbitrap-MS platform.

## 2. Results and Discussion

### 2.1. Advantages for UHPLC/Q-Orbitrap-MS Based Untargeted Profiling by the Enablinig of Rapid Polarity-Switching (ESI−/ESI+)

Studies have indicated that the combined use of negative and positive ESI modes may expand the coverage and provide different fragmentation information [19,20,21]. However, the rapid polarity switching by one injection analysis is not easy to be implemented on many high-resolution LC-MS platforms. Q-Orbitrap from Thermo Fisher Scientific can enable high-resolution MS^2^ determination by recording both the negative and positive data through once injection because of its capacity of rapid polarity switching. By applying it to the multicomponent characterization of EZP, both the negative and positive MS^2^ data were acquired in an untargeted DDA mode after a 25 min chromatographic separation. Utilization of the rapid polarity (ESI−/ESI+) switching UHPLC/Q-Orbitrap-MS displayed superiority over the single use of negative ESI detection (such as the method reported in literature [18]) in two aspects.

**Figure 2:**
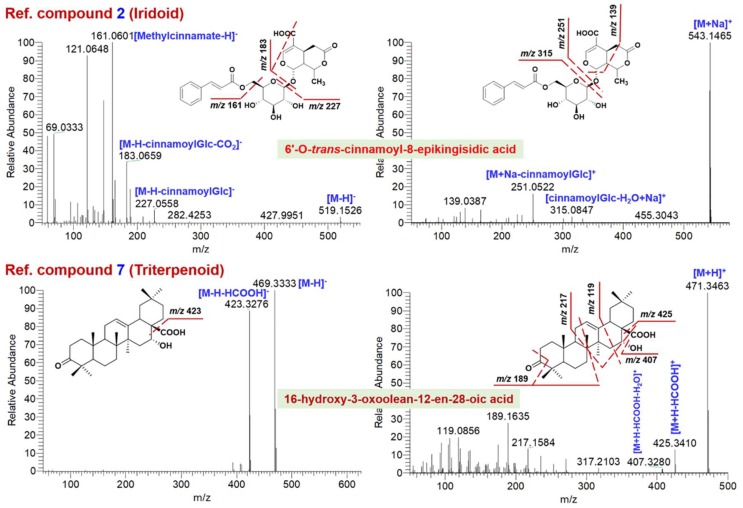
Annotation of the HCD-MS^2^ data for reference compounds **2** (Iridoid) and **7** (Triterpenoid) obtained in both negative and positive ESI modes.

**Figure 3:**
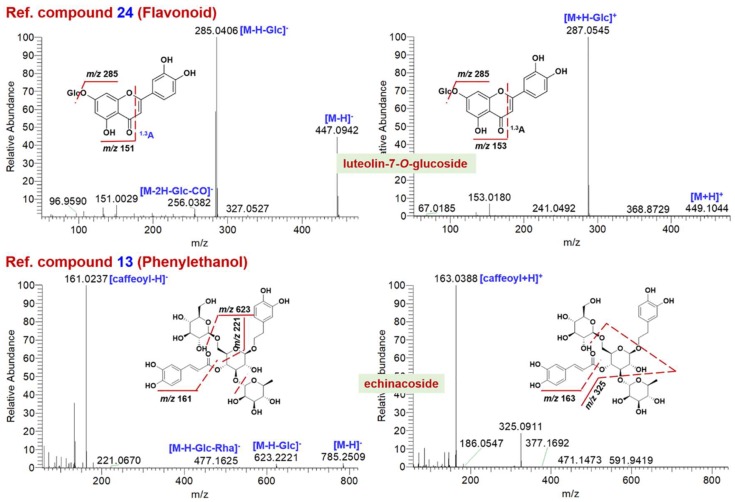
Annotation of the HCD-MS^2^ data for reference compounds **24** (Flavonoid) and **13** (Phenylethanol) obtained in both negative and positive ESI modes.

The MS^2^ data obtained in two ESI modes were complementary that rendered the structures elucidated more reliable. In the cases of 30 reference compounds (Figure 1), **11** could be detected from EZP in both two modes, while **8** and **5** compounds were only detected in ESI− or ESI+ mode (Table S1), respectively. It implied the significantly differential ionization behaviors for multiple components present in EZP. Figure 2 and Figure 3 exhibit annotation of the HCD-MS^2^ spectra for four representative reference compounds (**2**, **7**, **24**, and **13**) that could be well ionized by both ESI− and ESI+ modes, and evidently, the application of two ESI modes could produce more fragmentation information beneficial to their structural elucidation.

The coverage on EZP components was greatly improved when two ESI modes were applied. Some components poorly ionized in ESI− showed intense signals and thus could be characterized based on their positive HCD-MS^2^ data. In this work, 225 components (Tables S2 and S3) were only detectable in ESI+, and 18 thereof got tentatively characterized. It could indicate a significant improvement by approximately 2.6-fold in coverage for polarity-switching monitoring, compared with the single use of ESI− fragmentation in which 141 components were detected.

By the aforementioned advantages in combination with the high analysis efficiency facilitated by UHPLC separation, a powerful qualitative approach was established and applied to comprehensively profile multicomponents from EZP.

### 2.2. Comprehensive Characterization of the Multicomponents from EZP

Thirty compounds ever isolated from LLF and EH (Figure 1) were used as the reference to enhance reliability in multicomponent characterization. They structurally belong to seven different subclasses of botanical secondary metabolites (iridoid, **1**–**6**; triterpene, **7**–**12**; phenylethanol, **13**; coumarin, **14**–**16**; flavonoid, **17**–**27**; organic acid (ester), **28**–**29**; thiophene, **30**). Aside from the thiophenes, the others could be detected from EZP. Multiple approaches, involving elemental composition analysis, fragmentation pathways interpretation, retrieval of an in-house EZP library and the available chemistry database (like ChemSpider and PubChem, etc), were utilized to characterize those unknown components detected from EZP. Surprisingly, 366 compounds were separated and detected, and 96 of them (Table S2) were identified or tentatively characterized based on analyses of their negative and positive HCD-MS^2^ data. Notably, we used Glc to depict all the hexose residues for convenient expressions in this work.

**Figure 4:**
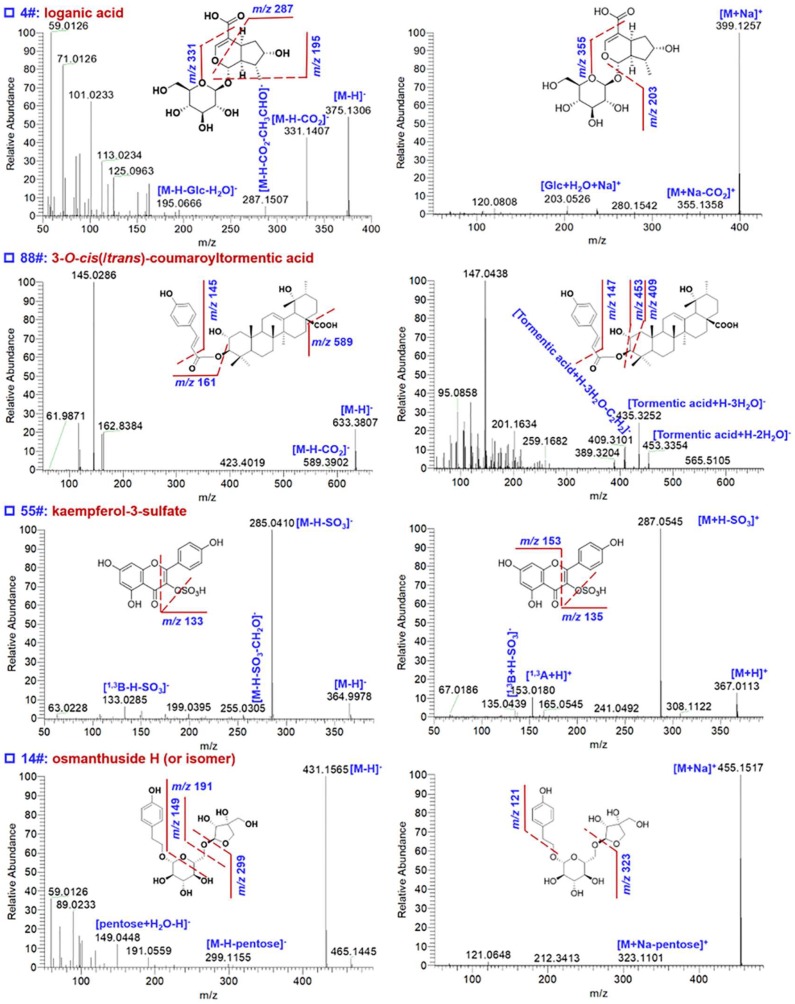
Fragmentation pathway analysis of four unknown compounds (**4**#: iridoid; **88**#: triterpenoid; **55**#: flavonoid; **14**#: phenylethanol) profiled from EZP based on the negative and positive HCD-MS^2^ data.

Iridoids detected from EZP were from LLF [18,22]. The fragmentation pathways of six iridoids (**1**–**6**, Figure 1) were comparatively studied, with reference compound **2** (6′-*O*-*trans*-cinnamoyl-8-epikingisidic acid) as an example illustrated in Figure 2. Rich deprotonated (*m*/*z* 519.1508) and sodium-adduct (*m*/*z* 543.1473) precursors were generated in ESI− and ESI+, respectively. Complementary fragmentation information could be obtained by HCD-MS^2^ in two ESI modes. Deprotonated molecules could be dissociated into the product ions at *m*/*z* 227.0558 and 183.0695 at medium intensity due to the neutral eliminations of cinnamoylglucose (cinnamoylGlc) and cinnamoylglucose plus CO_2_, while a base-peak fragment at *m*/*z* 161.0601 was assigned as deprotonated methylcinnamate. However, two complementary sodium-adduct product ions, because of the cleavage of ether bond between glucose and the iridoid framework, were obtained at *m*/*z* 315.0847 and 251.0522 by positive HCD-MS^2^. Additionally, a medium-intensity product ion of *m*/*z* 139.0387 was observed due to multiple bond fragmentation of the iridoid framework. In the case of an unknown iridoid compound (**4**# in Table S2; t_R_ 4.29 min), (Figure 4), the precursor ions in ESI− (*m*/*z* 375.1306) and ESI+ (*m*/*z* 399.1257) could infer the molecular formula C_16_H_24_O_10_. Diverse product ions in the negative mode, *m*/*z* 331.1407 ([M-H-CO_2_]^−^), 287.1507 ([M-H-CO_2_-CH_3_CHO]^−^), 195.0666 ([M-H-Glc-H_2_O]^−^), together with two positive fragments *m*/*z* 355.1358 ([M + Na-CO_2_]^+^) and 203.0526 ([Glc + H_2_O + Na]^+^), could infer the structure of loganic acid [23]. We could finally characterize 35 iridoid compounds from EZP, and six of them (**28**#: ligulucidumoside C: **39**#: oleuropeinic acid; **46**#: specnuezhenide; **65**#: oleonuezhenide; **70**#: 6′-*O*-*trans*-cinnamoyl-8-epikingisidic acid; **75**#: ligulucidumoside A; Table S2) had been confirmed with the aid of reference compounds.

Triterpenes and their glycosides (saponins) were the common bioactive components for LLF [22] and EH [24], and six triterpene compounds (**7**–**12**, Figure 1) were utilized as the reference compounds. Generally, MS^2^ fragmentation of free triterpenes were difficult to occur under the current MS condition (normalized collision energy, NCE; at 40%), resulting rare product ions. In contrast, the positive HCD-MS^2^ could produce diverse fragments as a result of ring cleavages on the skeleton. Reference compound **7** (16-hydroxy-3-oxoolean-12-en-28-oic acid) gave rich deprotonated precursors at *m*/*z* 469.3333 and protonated ones at *m*/*z* 471.3463 in ESI− and ESI+, respectively (Figure 2). Only one product ion (*m*/*z* 423.3276) by neutral elimination of HCOOH was readily obtained by negative MS^2^ fragmentation. However, much more diversified fragments (in addition to [M + H-HCOOH]^+^ detected at *m*/*z* 425.3410) could be dissociated from the protonated precursors in ESI+ mode. Fragmentations on ring C (along with neutral elimination of HCOOH or HCOH) could produce two product ions at *m*/*z* 217.1584 and 189.1635, while the ion of *m*/*z* 119.0856 should be a product fragment due to ring D fragmentation. Therefore, the fragments obtained in ESI+ could be more important for characterizing a free triterpene compound. Taking an unknown triterpenoid compound, compound **88**# (Table S2; t_R_ 18.14 min) as an example, rare product ions (neutral loss of CO_2_ and the coumaroyl substituent fragments) were dissociated from the deprotonated precursor of *m*/*z* 633.3807, and in contrast, rich product ions associated with the core triterpene structure were observed by HCD-MS^2^ of the protonated precursor at *m*/*z* 635.3942 (Figure 4). This evidence could assist in characterizing compound **88**# as 3-*O*-*cis* (or *trans*)-coumaroyltormentic acid, a known triterpenoid isolated from LLF [22]. According to these analyses, totally 18 triterpenoid compounds were characterized from EZP (Table S2), of which five (**81**#: ecliptasaponin A/D; **89**#: 16-hydroxy-3-oxoolean-12-en-28-oic acid; **90**#: echinocystic acid; **91**#: oleanolic acid; **96**#: 3β-*O*-acetylpomolic acid; Table S2) could be identified with high confidence because of reference compounds comparison.

Flavonoids are another category of common bioactive ingredients for LLF [22] and EH [24]. Up to eleven compounds (**17**–**27**, Figure 1) of this category had been taken as the reference compounds. Flavonoids (involving the free flavonoids and their *O*-glycosides) could be readily ionized by both the negative and positive ESI modes yielding diverse MS^2^ product ions that were diagnostic for their structural elucidation. The neutral loss resulting from the transition of precursors to aglycones were useful in characterizing the glycosyl moieties [25], while the secondary product ions of aglycones (particularly the RDA fragments) could be vital to identify the structures of flavonoid aglycones [26]. For instance, the reference compound **24** (luteolin-7-*O*-glucoside) gave parent ions at *m*/*z* 447.0942 ([M-H]^−^) and 449.1044 ([M + H]^+^) in ESI− and ESI+, respectively (Figure 3). The richest HCD-MS^2^ product ions obtained in two modes were both the aglycone product ions (*m*/*z* 285.0406 for ESI− and 287.0545 for ESI+). Differently, homolytic cleavage of the *O*-glycosidic bond could easily occur yielding the typical [Y_0_-H]^−^ ion at *m*/*z* 284.0332 (55% intensity of the Y_0_ ion) together with its secondary product ion because of the neutral loss of CO at *m*/*z* 256.0328 [13,25]. In addition, the ^1,3^A fragments in ESI− and ESI+ were observed at *m*/*z* 151.0029 and 153.0180, respectively. An unknown compound **55**# (Table S2; t_R_ 11.18 min) was speculated to be a flavonoid sulfate. The MS^2^ fragmentation in both two modes were easy to eliminate the neutral fragment SO_3_ forming the base-peak product ions (Figure 4). The fragment at *m*/*z* 255.0305 ascribed to [M-H-SO_3_-CH_2_O]^−^ could help identify this compound as a sulfate of kaempferol (flavonol) rather than luteolin (flavone) [25]. This structure could be further supported by RDA fragmentations (^1,3^B in ESI−; ^1,3^A and ^1,3^B in ESI+), and it was finally characterized as kaempferol 3-sulfate. Consequently, 15 flavonoid compounds were characterized from EZP (Table S2), nine of which (**11**#: skullcapflavone II; **25**#: 4′,7-dihydroxyl-3′,6′-dimethoxylisoflavone-7-*O*-glucoside; **41**#: luteolin-7-*O*-glucoside; **49**#: apigenin-7-*O*-glucoside; **53**#: acacetin; **56**#: kaempferol; **63**#: apigenin; **64**#: luteoline: **69**#: acacetin-7-*O*-rutinoside; Table S2) were compared with the reference compounds.

Phenylethanol compounds have been demonstrated as a class of bioactive ingredients for LLF [22]. In this work, seven phenylethanol compounds (Table S2) were characterized from EZP. Compound **31**# (t_R_ 8.00 min) was identified as echinacoside by comparison with the reference compound. Its negative HCD-MS^2^ spectrum displayed weak product ions due to successive neutral elimination of Glc (*m*/*z* 623.2221) and Rha (*m*/*z* 477.1625), together with a fragment of the terminal Glc (*m*/*z* 221.0670) and base-peak ion at *m*/*z* 161.0237 ascribed to the deprotonated caffeoyl group (Figure 3). Consistently, protonated caffeoyl ion at *m*/*z* 163.0388 and a medium-intensity product ion at *m*/*z* 325.0911 due to complex three-bond cleavage were dissociated from the precursor ion. For the unknown phenylethanol compound **14**# (t_R_ 5.48 min), a pentose (*m*/*z* 299.1155 for [M-H-pentose]^−^ and 323.1101 for [M + Na-pentose]^+^) and a hydroxyphenylethanol group (*m*/*z* 121.0648) attached to Glc could be characterized (Figure 4). Accordingly, we could characterize compound **14**# as osmanthuside H (or isomer) [23].

Additionally, 3 coumarin compounds, 20 phenols, and 8 miscellaneous were characterized from EZP, with their MS information listed in Table S2. It is noted that, the MS^2^ fragmentation information of much more components (particularly in the positive ESI mode) has been acquired. However, because of the unavailability of sufficient reference compounds, their structures could not be elicited only by the obtained MS data, and these compounds were regarded as unknown listed in Table S3. Their origin (LLF, EH, or the excipients used for preparing EZP) and structures would be studied in our future work.

It is noted that, despite the remarkable superiority in efficiency, the structural identification results (for those without reference comparison) obtained by the established LC-HRMS strategy are primary and tentative, which fail to exactly discriminate the sugars (such as glucose and galactose) and their stereochemistry (α- or β-configuration) and the glycosylation sites. Full establishment of their structures should be based on NMR and other spectroscopic analyses of pure isolated compounds.

### 2.3. Establishment of the ‘Identity Markers’ for EZP by Fingerprint Analysis

The quality standard of EZP recorded in *Chinese Pharmacopoeia* (2015 edition), which utilizes microscopic features examination (to identify LLF) and TLC comparison with the reference drug (to identify EH) [16], can hardly ensure the authenticity of EZP due to the insufficiency of specificity. Here we aimed to qualitatively authenticate EZP by the simultaneous monitoring of multiple quality markers, a solution to the authentication of TCM formulae that had been proven with the capacity of accurate identification and even discriminating easily confusing varieties [3,17]. For this purpose, the ‘Identity Markers’ of EZP were elaborated in the first step.

**Figure 5:**
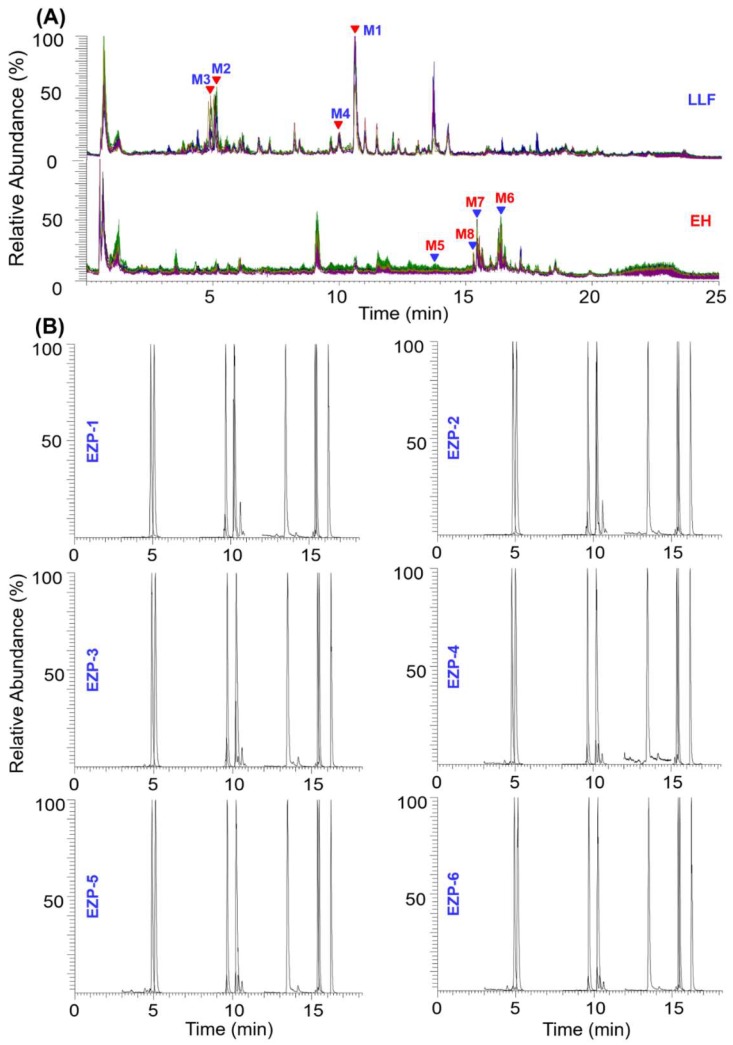
Overlapped fingerprints of two component drugs Ligustri Lucidi Fructus (LLF) and Ecliptae Herba (EH) under the same UHPLC/Q-Orbitrap MS condition (full scan in ESI− mode; **A**) and the SIM spectra of six batches of commercial EZP samples (**B**).

Fingerprint analyses of two component drugs LLF and EH (prepared according to their procedures used to produce EZP) were performed under the same LC-MS condition as that applied to capture the fingerprint of EZP (Table S4). Those rich and common compounds (bioactive) in multiple samples were selected as potential quality markers [3]. Figure 5 displays the fingerprints of wine-processed LLF (six batches, Table S4) and the decoction of EH (six batches). In contrast, the chemical profiles of LLF were similar, while EH showed larger difference in respect of batch-to-batch chemical consistency. Specnuezhenide (**M1**, Figure 5), an iridoid compound with the therapeutic potential for osteoarthritis [27] and diabetic retinopathy [28], currently is the unique quality marker for LLF and EZP as recorded in *Chinese Pharmacopoeia* (2015 version). It was a rich and common compound for LLF (Figure 5), and thus selected. In addition, 10-hydroxyoleoside dimethylester (iridoid, **M2**), salidroside (**M3**) and verbascoside (**M4**, two phenylethanol glycosides), were also chosen due to their high contents in LLF and well-reported bioactivities [23,29]. These four compounds constituted the markers for identifying LLF from EZP. On the other hand, despite wedelolactone (**M5**, coumarin) showed low extraction efficiency by water decocting (high-concentration ethanol benefits its extraction [30]), it is the only marker of EH recorded in *Chinese Pharmacopoeia* and could be detected from EZP [18]. Considering triterpenoids are the major bioactive ingredients for EH [24,31], three saponins including ecliptasaponin A/D (**M6**, co-eluting under the current UHPLC condition), eclalbasaponin C or isomer (**M7**), and eclalbasaponin VI (**M8**), which gave intense peaks in TICs of EH decoction, were also considered as the markers of EH. By these analyses, as a result, the ‘identity markers’ of EZP were established that involved these eight compounds.

### 2.4. Authentication of Commercial EZP Samples by Simultaneously Monitoring Eight ‘Identity Markers’ Using UHPLC/Q-Orbitrap-MS Based SIM Approach

To intuitively and sensitively monitor the deduced ‘identity markers’ in real, commercial EZP samples (presence or absence), an SIM method was developed on the Q-Orbitrap mass spectrometer using the Target SIM scan method [3,32]. All eight marker compounds were detected in ESI− mode with SID set at 5 eV (*m*/*z* 685.2358 for specnuezhenide, t_R_ 5.04 min; 433.0996 for 10-hydroxyoleoside dimethyl ester, t_R_ 5.04 min; 299.1140 for salidroside, t_R_ 4.82 min; 623.1993 for verbascoside, t_R_ 9.80 min; 313.0363 for wedelolactone, t_R_ 13.83 min; 633.4014 for ecliptasaponin A/D, t_R_ 16.23 min; 841.4614 for eclalbasaponin C (or isomer), t_R_ 15.39 min; and 875.4130 for eclalbasaponin VI, t_R_ 15.33 min). The obtained SIM spectra for six batches of EZP samples (Table S4), EZP-1 to EZP-6 (collected from three different vendors: Tianjin Zhongxin Pharmaceutical, Jiangxi Renfeng Pharmaceutical, and Jiangxi Yaodu Zhangshu Pharmaceutical) are exhibited in Figure 5.

As shown in the SIM spectra, it was easy and convenient to distinguish the presence or absence of eight deduced marker compounds (t_R_ and MS information) in real EZP samples. Consequently, all these ‘identity markers’ could be observed for six batches of EZP samples we had analyzed, which could primarily testify their authenticity. Moreover, despite being from different vendors, the SIM profiles were very similar. The quality differentiation of EZP due to the different vendors could not be simply concluded only by the qualitative identification experiment, and necessary quantitative assays of these ‘identity markers’ together with some other bioactive components would be conducted in the next stage of our research work. It proves that, UHPLC coupled with SIM established on Q-Orbitrap-MS is a powerful vehicle that simultaneously identifies multiple drugs to enable the authentication of TCM formulae more specific and more reliable, in contrast to the approaches utilized in *Chinese Pharmacopoeia*. Moreover, the highly specific SIM method for authentication of TCM can be transformed onto any quadrupole containing mass spectrometers to expand the practicability in routine TCM analysis and quality examination.

## 3. Materials and Methods

### 3.1. Reagents and Chemicals

Thirty compounds (Figure 1), involving six iridoids (**1**: oleonuezhenide; **2**: 6′-*O*-*trans*-cinnamoyl-8-epikingisidic acid; **3**: ligulucidumoside C; **4**: ligulucidumoside A; **5**: oleuropeinic acid; **6**: specnuezhenide), six triterpenoids (**7**: 16-hydroxy-3-oxoolean-12-en-28-oic acid; **8**: 3β-*O*-acetylpomolic acid; **9**: ecliptasaponin A; **10**: ecliptasaponin D; **11**: echinocystic acid; **12**: oleanolic acid), one phenyletanol (**13**: echinacoside), three coumarins (**14**: psoralen; **15**: isopsoralen; **16**: wedelolactone), eleven flavonoids (**17**: apigenin; **18**: luteoline; **19**: kaempferol; **20**: quercetin; **21**: acacetin; **22**: acacetin-7-*O*-rutinoside; **23**: apigenin-7-*O*-glucoside; **24**: luteolin-7-*O*-glucoside; **25**: kaempferol-4′-methyl ether; **26**: skullcapflavone II; **27**: 4′,7-dihydroxyl-3′,6′-dimethoxylisoflavone-7-*O*-glucoside), two organic acids (esters) (**28**: ethyl caffeate; **29**: ethyl protocatechuate), and one thiophene (**30**: a-formylterthienyl), were used as the reference compounds in this study. Amongst them, **2**–**5**, **7**, **8**, **18**, **22**–**25**, **27**, and **30**, were isolated from LLF or EH [33,34], while the others were purchased from Shanghai Standard Biotech. Co., Ltd. (**1**, **6**, **9**–**17**, **19**–**21**, and **28**) or Shanghai Yuanye Biotech. Co., Ltd. (**26** and **29**) (Shanghai, China). More information of these reference compounds is given in Table S1. HPLC-grade acetonitrile, methanol (Fisher, Fair lawn, NJ, USA), formic acid (Sigma-Aldrich, Louis, MO, USA), and ultra-pure water in-house prepared using a Milli-Q water purification system (Millipore, Bedford, MA, USA), were used. Detailed information with respect to multiple batches of LLF and EH samples, as well as EZP, is provided in Table S4.

### 3.2. Sample Preparation

An easy-to-implement ultrasound-assisted extraction method was utilized in sample preparation of EZP. Briefly, 10 mg accurately weighed powder of EZP was soaked in 20 mL 50% aqueous methanol (*v*/*v*). After vortex mixing for 2 min, the liquid was extracted on a water bath (Millipore, Bedford, MA, USA) with ultrasound aiding for 1 h. After a 10 min centrifugation (Eppendorf 5804R, Eppendorf AG, Hamburg, Germany) at 14,000 rpm, the supernatant was taken as the test solution of EZP for multicomponent characterization and authentication assays (10 mg/mL). For fingerprint analyses, the samples of LLF and EH were prepared mainly following the methods of *Chinese Pharmacopoeia* (2015 version) described for EZP. In detail, accurately weighed LLF (wine-processed) powder (100 mg) was ultrasonically extracted with 10 mL 50% methanol for 40 min. The same centrifuging condition was applied to yield the supernatant as the test solution of LLF (10 mg/mL). For EH, 100 g powder was decocted with water twice (1 h for each time; 2 L and 1 L water separately used). The pooled decoction was filtered and further concentrated to dryness under reduced pressure. An aliquot of EH extract equivalent to 100 mg of EH was accurately weighed and dissolved with 10 mL 50% methanol. The supernatant obtained after centrifugation was used as the test solution for EH.

### 3.3. Chromatographic Separation and MS Conditions

Rapid chromatographic separation was achieved on an Ultimate 3000 UHPLC system (Thermo Fisher Scientific, Waltham, MA, USA) configured with a BEH C18 column (2.1 × 100 mm, 1.7 µm; Waters Co., Milford, MA, USA) maintained at 25 °C. The mobile phase consisted of water containing 0.1% formic acid (A) and acetonitrile (B) and ran in accordance with an optimal gradient program: 0–13.5 min, 1–30% (B); 13.5–14 min, 30–32% (B); 14–14.5 min, 32–45% (B); 14.5–16.5 min, 45–64% (B), 16.5–21.5 min, 64–100% (B); and 21.5–26.5 min, 100% (B). The flow rate was set at 0.4 mL/min. Both the fingerprint analysis of two component drugs and the multicomponent characterization of EZP experiments were conducted following the same UHPLC condition. The injection volume for all samples (LLF, EH, and EZP) was 5 µL.

HRMS data were recorded on a Q ExactiveTM hybrid Q–Orbitrap mass spectrometer equipped with a heated ESI source (Thermo Fisher Scientific). The ESI source parameters were set as follows: spray voltage, −2.5 kV/3.0 kV; sheath gas pressure, 40 arb; aux gas pressure, 10 arb; sweep gas pressure, 0 arb; capillary temperature, 350 °C; and aux gas heater temperature, 400 °C. In the experiments of multicomponent characterization of EZP, a Full MS/dd-MS^2^ (TopN) scan method was used. The Orbitrap analyzer scanned over a range of *m*/*z* 100–1500 at a resolution of 70,000 in full scan MS^1^, and a resolution of 13,500 for data-dependent MS^2^. AGC target for MS^1^ and MS^2^ was set at 5e6 and 1e5, respectively. Maximum IT for MS^1^ and MS^2^ scans was separately defined at 150 ms and 100 ms. MS/MS experiments were performed by HCD at NCE combination 30/40/50 V, with an isolation width of 4.0 Da. An Apex trigger of 2–6 s was enabled to acquire the MS^2^ fragments of precursors at the highest abundance. Exclusion time 6 s was set for dynamic exclusion that could trigger the acquisition of MS^2^ fragments of more minor components in case co-elution occurred.

In the authentication of EZP, Target SIM experiment was conducted that could enable the selective monitoring of eight ‘identity markers’ of EZP: *m*/*z* 685.2358 for specnuezhenide (C_31_H_42_O_17_), *m*/*z* 433.0996 for 10-hydroxyoleoside dimethyl ester (C_18_H_26_O_12_), *m*/*z* 299.1140 for salidroside (C_14_H_20_O_7_), *m*/*z* 623.1993 for verbascoside (C_29_H_36_O_15_), *m*/*z* 633.4014 for ecliptasaponin A (C_36_H_58_O_9)_, *m*/*z* 841.4614 for ecliptasaponin V (C_42_H_66_O_17_), *m*/*z* 875.4130 for ecliptasaponin VI (C_42_H_68_O_17_S), and *m*/*z* 313.0363 for wedelolactone (C_16_H_10_O_7_).

## 4. Conclusions

Aiming to comprehensively elucidate the chemical constituents and conveniently perform the authentication of TCM formulae, two different approaches were established by taking advantages of the UHPLC/Q-Orbitrap-MS platform with EZP as a case. An untargeted dd-MS^2^ method by enabling rapid polarity switching between ESI− and ESI+ was developed, which improved the coverage of EZP component by approximately 2.6-fold, compared with the single use of negative mode. It could enable the separation and detection of as many as 366 components from EZP by one injection analysis, and 96 thereof were identified or primarily characterized by analyzing the negative and positive HCD-MS^2^ data. By fingerprint analyses of LLF (wine-processed) and EH (water decoction) under the same condition, eight compounds were finally selected as the ‘identity markers’ for identification of EZP. SIM spectra could intuitively embody the presence of LLF and EH markers in EZP. Six batches of EZP samples from three vendors showed good consistency. Conclusively, comprehensive chemical profiling and reliable authentication of EZP were accomplished. UHPLC/Q-Orbitrap-MS is a powerful platform that can facilitate the in-depth chemical profiling and authentication of TCM formulae.

## Supplementary Materials

The following are available online: Table S1: Detailed information of 30 reference compounds and their MS data acquired from the EZP sample by UHPLC/Q-Orbitrap-MS; Table S2: Detailed information of the 96 components characterized from EZP; Table S3: Information of the 270 components failing to be characterized from EZP; Table S4: Information of the LLF, EH, and EZP samples analyzed in this work.
