# Supplementary material for: Systematic Profiling of the Multicomponents and Authentication of Erzhi Pill by UHPLC/Q-Orbitrap-MS Oriented Rapid Polarity-Switching Data-Dependent Acquisition and Selective Monitoring of the Chemical Markers Deduced from Fingerprint Analysis
Source: Molecules. 2018 Nov 30;23(12):3143. doi: 10.3390/molecules23123143 (PMC6320785; doi:10.3390/molecules23123143)
Supplement: Supplementary file 1 [file molecules-23-03143-s001.pdf]

<*Molecules*>

SUPPLEMENTARY MATERIALS

Systematic profiling of the multicomponents and authentication of Erzhi Pill by UHPLC/Q-Orbitrap-MS oriented rapid polarity-switching data-dependent acquisition and selective monitoring of the chemical markers deduced from fingerprint analysis

Contents

Table S1 Detailed information of 30 reference compounds and their MS data acquired from the EZP sample by UHPLC/Q-Orbitrap-MS.

Table S2 Detailed information of the 96 components characterized from EZP.

Table S3 Information of the 270 components failing to be characterized from EZP.

Table S4 Information of the LLF, EH, and EZP samples analyzed in this work.

Table S1 Detailed information of 30 reference compounds and their MS data acquired from the EZP sample by UHPLC/Q-Orbitrap-MS.

No.	Compound	Formula	Exact Mass	Structure Subclass	ESI-: MS^1	ESI-: MS^2	ESI+: MS^1	ESI+: MS^2
**1**	oleonuezhenide	$C_{48}H_{64}O_{27}$	1072.3635	Iridoid	1071.3562	839.2621, 753.0600, 685.2361, 653.2118, 523.1832, 453.1414, 421.1520, 299.1142, 121.0285	1090.3973	695.2276, 611.3927, 369.1171, 225.0750, 165.0542
**2**	6'-*O*-*trans*-cinnamoyl-8-epikingisidic acid	$C_{25}H_{28}O_{12}$	520.1575	Iridoid	519.1508	519.1526, 427.9951, 282.4253, 227.0558, 183.0659, 161.0601	543.1473	543.1456, 455.3043, 315.0847, 251.0522, 225.0754, 139.0387
**3**	ligulucidumoside C	$C_{19}H_{26}O_{13}$	462.1373	Iridoid	461.1323	341.0462, 297.0988, 281.0677, 255.0878, 237.0769, 191.0347, 176.0110, 149.0236, 113.0233, 101.0234	485.1266	485.1256, 368.1511, 323.0733, 291.0469, 261.0720, 164.0698
**4**	ligulucidumoside A	$C_{26}H_{34}O_{12}$	538.2050	Iridoid	-	-	556.2389	555.2753, 489.1753, 369.1176, 295.0803, 225.0753, 193.0487, 165.0544, 151.0387
**5**	oleuropeinic acid	$C_{25}H_{30}O_{15}$	570.1585	Iridoid	569.1540	569.1566,389.0882, 363.0882,363.1096, 345.0958,331.0833, 313.0721,299.1153, 281.1036,227.0561,	-	-

						221.0092,209.0454, 193.0139,183.0659, 177.0189,165.0551, 151.0394,133.0286, 123.0442, 101.0233		
**6**	specnuezhenide	$C_{31}H_{42}O_{17}$	686.2422	Iridoid	685.2349	523.1824, 453.1409, 421.1512, 299.1147, 271.9377, 223.0614, 179.053, 121.0284	704.2734	475.1559, 369.1174, 295.0807, 225.0753, 165.0544, 151.0388
**7**	16-hydroxy-3-oxoolean-12-en-28-oic acid	$C_{30}H_{46}O_4$	470.3391	Triterpenoid	469.3340	469.3333, 423.3276, 393.2817, 159.4625	471.3459	471.3463, 425.3410, 407.3280, 317.2103, 235.1691, 217.1584, 189.1635, 119.0856
**8**	3*β*-*O*-acetylpomolic acid	$C_{32}H_{50}O_5$	514.3658	Triterpenoid	-	-	515.3720	485.3616, 469.3670, 437.3397, 373.2734, 301.2154, 255.2095, 215.1792, 189.1636, 119.0856
**9**	ecliptasaponin A	$C_{36}H_{58}O_9$	634.4081	Triterpenoid	633.4008	633.4017, 491.1723, 231.8210, 161.0442, 113.0234, 101.0232	-	-
**10**	ecliptasaponin D	$C_{36}H_{58}O_9$	634.4081	Triterpenoid	-	-	-	-
**11**	echinocystic acid	$C_{30}H_{48}O_4$	472.3553	Triterpenoid	471.3496	471.3491, 453.3375, 340.7722, 224.6396, 142.4072,	473.3614	473.3626, 437.3397, 427.3564, 409.3457, 331.2617, 255.2107, 201.1635, 133.1012

**12**	oleanolic acid	$C_{30}H_{48}O_3$	456.3603	Triterpenoid	-	-	457.3665	457.3669, 439.3582, 410.3492, 303.2322, 221.1897, 189.1635, 135.1167, 121.1013
**13**	echinacoside	$C_{35}H_{46}O_{20}$	786.2582	Phenylethanol	785.2510	785.2509, 748.8304, 744.3573, 654.5068, 623.2221, 532.9862, 477.1625, 460.4425, 445.6915, 432.9994, 376.8912, 315.1069, 261.0775, 221.0670, 179.0345, 161.0237, 133.0285, 113.0233	804.2895	591.9419, 471.1473, 325.0911, 186.0547, 163.0388
**14**	psoralen	$C_{11}H_6O_3$	186.0317	Coumarin	-	-	-	-
**15**	isopsoralen	$C_{11}H_6O_3$	186.0317	Coumarin	-	-	-	-
**16**	wedelolactone	$C_{16}H_{10}O_7$	314.0427	Coumarin	313.0354	313.0363, 298.0125, 283.0252, 268.0019, 240.0067, 212.0114, 186.0321, 132.0193, 109.0269	-	-
**17**	apigenin	$C_{15}H_{10}O_5$	270.0528	Flavonoid	269.0455	269.0461, 225.0555, 183.0444, 159.0444, 117.0335	271.0601	271.0598, 225.0908, 178.0777, 153.0180, 119.0492
**18**	luteoline	$C_{15}H_{10}O_6$	286.0477	Flavonoid	285.0411	285.0410, 267.0306, 241.0514, 199.0397, 175.0395, 151.0029,	287.0546	287.0545, 269.0435, 241.0494, 213.0539, 161.0230, 153.0181,

						133.0285, 107.0127		135.0440
**19**	kaempferol	$C_{15}H_{10}O_6$	286.0477	Flavonoid	285.0405	285.0410,283.0248, 267.0287,255.0304, 243.0304,229.0522, 211.0401,199.0398, 185.0601,175.0397, 159.0451,151.0028, 147.0448,133.0286, 121.0285, 107.0128	-	-
**20**	quercetin	$C_{15}H_{10}O_7$	302.0427	Flavonoid	-	-	-	-
**21**	acacetin	$C_{16}H_{12}O_5$	284.0685	Flavonoid	-	-	302.1019	302.1020, 270.1121, 256.0963, 242.0803, 199.0625, 164.0704, 138.0550, 108.0445
**22**	acacetin-7-*O*-rutinoside	$C_{28}H_{32}O_{14}$	592.1792	Flavonoid	-	-	593.1855	394.7499, 319.0821, 277.0704, 183.0649, 165.0544, 151.0388
**23**	apigenin-7-*O*-glucoside	$C_{21}H_{20}O_{10}$	432.1056	Flavonoid	431.0984	431.0990, 395.9469, 311.0600, 268.0382, 240.0425, 151.0028, 107.0127	433.1116	433.1132, 358.6413, 271.0595, 225.0546, 153.0180, 119.0492
**24**	luteolin-7-*O*-glucoside	$C_{21}H_{20}O_{11}$	448.1006	Flavonoid	447.0933	447.0942, 327.0527, 297.0417, 285.0406, 256.0382, 199.0394, 151.0029, 107.0127	449.1075	449.1044, 368.8729, 287.0545, 269.0444, 241.0492, 203.0346, 153.0180
**25**	kaempferol-4'-methyl ether	$C_{16}H_{12}O_6$	300.0634	Flavonoid	-	-	-	-

**26**	skullcapflavone II	$C_{19}H_{18}O_8$	374.1002	Flavonoid	373.0929	373.0775, 329.0887, 266.6675, 193.0140, 167.0343, 139.0391, 124.0155	-	-
**27**	4',7-dihydroxyl-3',6'-dimethoxylisoflavone-7-*O*-glucoside	$C_{23}H_{24}O_{11}$	476.1319	Flavonoid	475.1246	475.1121, 431.1186, 355.1075, 221.0091, 209.0455, 165.0551, 133.0285	-	-
**28**	ethyl caffeate	$C_{11}H_{12}O_4$	208.0736	Organic acid (ester)	207.0663	207.0660, 179.0344, 161.0237, 139.0391, 135.0442, 125.8722	-	-
**29**	ethyl protocatechuate	$C_9H_{10}O_4$	182.0579	Organic acid (ester)	181.0506	181.0501,179.8413, 167.9042,161.8754, 153.0186,145.8615, 143.8643,141.8674, 135.0078,124.0159, 121.0283,111.0077, 109.0284, 103.9191	-	-
**30**	a-formylterthienyl	$C_{13}H_8OS_3$	275.9737	Thiophene	-	-	-	-

-: not detected or not available.

Table S2 Detailed information of the 96 components characterized from EZP.

No.	t_R (min)	Parent ion (ESI-: MS^1/ ESI+: MS^1)	Mass error (ppm)	RDB	Formula	ESI-: MS^2	ESI+: MS^2	Identification	Structure Subclass
1	0.81	195.0509/-	-1.415	1.5	$C_6H_{12}O_7$	195.0508, 193.0592, 177.0398, 171.1603, 159.0293, 150.5823, 129.0184, 125.4034, 120.8055, 111.0078, 101.0232, 99.0077	-	D-gluconic acid	others
2	3.63	153.0188/-	-4.979	5.5	$C_7H_6O_4$	153.0188, 151.0392, 142.9455, 135.0445, 126.9027, 123.0444, 114.9510, 110.0317, 109.0285, 108.0207, 103.9194	-	protocatechuic acid	Phenolic acids
3	3.86	153.0553/-	-4.818	4.5	$C_8H_{10}O_3$	153.0550, 151.0392, 137.0233, 125.0494, 124.0476, 123.0442, 122.0364, 110.0317, 109.0284, 108.0207	-	hydroxytyrosol	Phenylethanols
4	4.29	375.1308/399.1254[a]	-1.173	4.5	$C_{16}H_{24}O_{10}$	375.1306, 331.1407, 287.1507, 195.0666, 162.8385, 125.0963, 101.0233	399.1257, 355.1358, 280.1542, 203.0526, 120.0808	loganic acid	Iridoid
5	4.35	451.1113/-	1.799	6.5	$C_{17}H_{24}O_{14}$	451.1101, 433.0984, 389.1105, 373.0799, 278.7802, 271.0464, 256.5335, 239.0202, 227.0563, 209.0456, 195.0297, 181.0501, 165.0552, 151.0394, 139.0393, 123.0442, 107.0492, 101.0233	-	nuezhenidic acid	Iridoids

6	4.43	353.0893/-	1.628	8.5	$C_{16}H_{18}O_9$	353.0884, 346.5439, 315.1101, 285.9144, 255.9048, 220.9500, 191.0558, 179.0345, 173.0450, 161.0238, 151.0393, 135.0443, 127.0390, 111.0440	-	neochlorogenic acid	Phenolic acids
7	4.46	241.0725/-	-0.379	5.5	$C_{11}H_{14}O_6$	241.0717, 239.0557, 224.1083, 214.0867, 209.0454, 206.8249, 191.0340, 181.0504, 179.0711, 165.0546, 160.8415, 151.0390, 149.0236, 147.0443, 135.0442, 127.0391, 123.0441, 121.0285, 111.0076, 103.9192	-	isomer of elenolic acid	Iridoids
8	4.65	213.0770/-	-0.313	4.5	$C_{10}H_{14}O_5$	213.0768, 211.0611, 197.0449, 186.8974, 171.0658, 168.8863, 159.9361, 157.8623, 153.0548, 144.0082, 139.0392, 137.0227, 127.0753, 125.0598, 122.8931, 121.0283, 111.0079, 103.9191	-	nuzhenal A or isomer	Iridoids
9	4.86	375.1308/-	3.092	5.5	$C_{16}H_{24}O_{10}$	375.1308, 348.2830, 331.1403, 287.1500, 213.0756, 198.4171, 195.0663, 191,0561, 179.0553, 162.8385, 161.0447, 151.0757, 143.0342, 139.0746, 125.0965, 123.0443, 119.0340, 113.0233, 101.0233	-	loganic acid or isomer	Iridoids
10	4.99	299.1147/-	-1.659	5.5	$C_{14}H_{20}O_7$	299.1131, 265.1093, 226.0034,	-	salidroside	Phenylethanols

						179.0559, 143.0342, 119.0492, 113.0232, 101.0233			
11[*]	5.27	373.0929/-	-0.495	7.5	$C_{19}H_{18}O_8$	373.0775, 329.0887, 266.6675, 193.0140, 167.0343, 139.0391, 124.0155	-	skullcapflavone II	Flavonoid
12	5.34	433.1008/457.0953[a]	4.84	7.5	$C_{17}H_{22}O_{13}$	433.1009, 401.0735, 389.1081, 349.0616, 271.0442, 227.0919, 221.0093, 209.0455, 195.0289, 183.0657, 177.0189, 165.0549, 149.0238, 133.0285, 125.0235, 105.0337, 101.0233	457.0941, 322.6229, 295.0418, 263.0158	10-hydroxyoleoside dimethylester	Iridoids
13	5.38	213.0770/-	-0.454	4.5	$C_{10}H_{14}O_5$	213.0768, 211.0617, 195.0652, 186.8973, 184.0976, 168.8865, 159.9565, 157.8623, 151.0757, 139.0758, 137.0591, 125.0599, 122.8932, 121.0280, 103.9190, 102.0550	-	nuzhenal A or isomer	Iridoids
14	5.48	431.1576/455.1524[a]	2.401	6.5	$C_{19}H_{28}O_{11}$	431.1569, 299.1155, 251.0790, 221.0093, 205.6825, 191.0557, 179.0551, 162.8388, 161.0447, 149.0448, 131.0336, 119.0491, 101.0233	455.1517, 323.1101, 212.3413, 121.0648	osmanthuside H or isomer	Phenylethanols
15	5.49	-/455.1511[a]	-1.566	5.5	$C_{19}H_{28}O_{11}$	-	455.1517, 323.1101, 212.3413, 121.0648	benzyl Gentiobioside or isomer	Iridoids
16	5.66	353.0892/-	2.76	8.5	$C_{16}H_{18}O_9$	353.0888, 191.0558, 173.0448, 161.0238, 137.0235, 113.0233	-	chlorogenic acid	Phenolic acids

17	5.91	377.1469/-	2.121	4.5	$C_{16}H_{26}O_{10}$	377.1461, 331.1410, 276.4047, 248.8959, 205.0496, 197.0817, 173.0456, 160.8412, 153.0916, 149.0230, 125.0234, 113.0234, 101.0233	-	a glucoside of $C_{10}H_{16}O_5$	others
18	6.05	353.0892/-	0.75	8.5	$C_{16}H_{18}O_9$	353.0881, 346.5798, 309.0989, 261.6462, 236.9430, 219.0677, 203.0348, 195.0305, 191.0557, 179.0344, 173.0449, 162.8389, 155.0341, 149.0603, 139.0385, 135.0442, 127.0388, 123.0441, 111.0440, 103.9191	-	cryptochlorogenic acid	Phenolic acids
19	6.16	389.1107/413.1054[a]	1.222	6.5	$C_{16}H_{22}O_{11}$	389.1094, 345.1199, 330.0663, 255.6959, 240.3965, 209.0449, 190.4853, 183.0657, 165.0547, 160.8416, 139.0028, 131.0347, 121.0648, 113.0233, 101.0233	413.1015, 369.1147, 307.3437, 251.0522, 233.0416, 120.0809	oleoside/ secologanoside or isomer	Iridoids
20	6.20	153.0190/-	-4.979	5.5	$C_7H_6O_4$	153.0186, 151.0393, 139.0394, 126.9027, 123.0443, 114.9506, 110.0318, 109.0285, 108.0207, 103.9192	-	protocatechuic acid or isomer	Phenolic acids
21	6.35	461.1326/-	4.762	7.5	$C_{19}H_{26}O_{13}$	461.1323, 429.1057, 405.0088, 385.1165, 299.1141, 245.0842, 237.0772, 223.0614, 205.0505, 193.0868, 175.0761, 161.0600, 143.0340, 127.0391, 109.0648,	-	sibiricose A3 or isomer	others

						101.0233			
22	6.87	357.1207/-	2.589	6.5	$C_{16}H_{22}O_9$	357.1200, 315.1094, 297.0988, 287.0176, 261.7538, 222.3102, 219.5779, 197.6418, 182.0219, 181.0502, 162.8386, 153.0546, 149.0595, 143.0341, 135.0442, 123.0442, 119.0337, 113.0235, 101.0233	-	sweroside or isomer	Iridoids
23	7.05	377.1469/401.1412[a]	2.359	4.5	$C_{16}H_{26}O_{10}$	377.1462, 338.1765, 299.0208, 197.0817, 185.2895, 169.0867, 153.0914, 146.4314, 135.0809, 119.0338, 101.0233	401.1412, 383.1305, 239.0887, 185.0418	oleuropein aglycone or isomer	Iridoids
24	7.12	181.0506/-	-2.11	5.5	$C_9H_{10}O_4$	196.7434, 182.8597, 181.0502, 179.0344, 166.0260, 163.0394, 159.8776, 152.9169, 138.0634, 137.0600, 135.0443, 124.0158, 122.0364, 119.0493, 109.0282, 103.91912	-	isomer of ethyl protocatechuate	Phenolic acids
25[*]	7.15	475.1246/-	4.655	8.5	$C_{23}H_{24}O_{11}$	475.1121, 431.1186, 355.1075, 221.0091, 209.0455, 165.0551, 133.0285		4',7-dihydroxyl-3',6'-dimethoxylisoflavone-7-*O*-glucoside	Flavonoid
26	7.35	-/413.1411[a]	-2.876	4.5	$C_{17}H_{26}O_{10}$	-	413.1406, 329.1504, 251.0890, 233.0779, 203.0525	secologanol or isomer	Iridoids

27	7.60	377.1471/-	0.106	4.5	$C_{16}H_{26}O_{10}$	377.1454, 359.0762, 337.9098, 318.0714, 299.0206, 263.0575, 235.0626, 197.0816, 189.0554, 162.8379, 153.0913, 147.0445, 119.0340, 101.0233	-	oleuropein aglycone or isomer	Iridoids
28[*]	7.71	461.1323/485.1266[a]	5.5	7.5	$C_{19}H_{26}O_{13}$	341.0462, 297.0988, 281.0677, 255.0878, 237.0769, 191.0347, 176.0110, 149.0236, 113.0233, 101.0234	485.1256, 368.1511, 323.0733, 291.0469, 261.0720, 164.0698	ligulucidumoside C	Iridoids
29	7.89	461.1324/-	3.917	7.5	$C_{19}H_{26}O_{13}$	461.1319, 425.9385, 403.1264, 323.9066, 300.0780, 281.0672, 223.0607, 205.0492, 179.0554, 160.8413, 147.0443, 113.0234, 101.0233	-	isomer of ligulucidumoside C	Iridoids
30	8.00	377.1462/-	1.644	4.5	$C_{16}H_{26}O_{10}$	377.1459, 244.0188, 197.0818, 184.7623, 153.0913, 119.0344, 101.0233	-	oleuropein aglycone or isomer	Iridoids
31[*]	8.00	785.2537/804.2895[b]	-0.149	13.5	$C_{35}H_{46}O_{20}$	785.2509, 748.8304, 744.3573, 654.5068, 623.2221, 532.9862, 477.1625, 460.4425, 445.6915, 432.9994, 376.8912, 315.1069, 261.0775, 221.0670, 179.0345, 161.0237, 133.0285, 113.0233	591.9419, 471.1473, 325.0911, 186.0547, 163.0388	echinacoside	Phenylethanols
32	8.19	-/427.1202[a]	-1.411	5.5	$C_{17}H_{24}O_{11}$	-	427.1205, 381.0873, 288.0844, 247.0575, 203.0524, 165.0543	secoxyloganin or isomer	others

33	8.24	241.0725/-	1.073	5.5	$C_{11}H_{14}O_6$	241.0720, 223.0690, 209.0452, 199.0609, 191.0345, 181.0500, 179.0708, 172.9400, 167.0343, 160.8416, 153.0548, 147.0440, 139.0028, 137.0600, 127.0391, 121.0285, 111.0078, 103.9192, 101.0233	-	elenolic acid	Iridoids
34	8.48	-/429.1363[a]	-2.593	4.5	$C_{17}H_{26}O_{11}$	-	429.1356, 397.1102, 257.0987, 136.0617	8-*O*-acetylharpagide or isomer	others
35	8.81	403.1264/-	1.477	6.5	$C_{17}H_{24}O_{11}$	403.1252, 371.0988, 359.1359, 327.1121, 298.0124, 283.0839, 277.0697, 253.0512, 241.0719, 223.0607, 209.0452, 197.0815, 181.0713, 179.0708, 165.0550, 160.8416, 149.0237, 147.0441, 139.0028, 127.0391, 121.0285, 111.0077, 101.0233	-	oleoside 11-methyl ester or isomer	Iridoids
36	9.03	403.1260/-	0.956	6.5	$C_{17}H_{24}O_{11}$	403.1250, 380.7274, 373.1147, 359.1358, 329.1266, 310.0684, 283.0818, 241.0719, 223.0623, 209.0451, 181.0715, 165.0551, 160.8412, 147.0445, 139.0029, 127.0391, 121.0285, 111.0077, 101.0233	-	oleoside 11-methyl ester or isomer	Iridoids

37	9.07	593.1536/-	3.181	13.5	$C_{27}H_{30}O_{15}$	593.1531, 398.1811, 373.9933, 297.0403, 285.0409, 256.0374, 217.0502, 199.0396, 151.0032, 133.0287, 107.0128	-	luteolin-O-rutinoside	Flavonoids
38	9.08	-/441.1360[a]	-2.794	5.5	$C_{18}H_{26}O_{11}$	-	441.1355, 279.0835, 247.0572, 205.0474, 165.0544, 121.0645	secoxyloganin methy l ester or isomer	Iridoids
39*	9.22	569.1540/-	-0.89	2.5	$C_{18}H_{34}O_{20}$	569.1566, 389.0882, 363.0882, 363.1096, 345.0958, 331.0833, 313.0721, 299.1153, 281.1036, 227.0561, 221.0092, 209.0454, 193.0139, 183.0659, 177.0189, 165.0551, 151.0394, 133.0286, 123.0442, 101.0233	-	oleuropeinic acid	Iridoids
40	9.25	701.2321/-	1.159	11.5	$C_{31}H_{42}O_{18}$	701.2307, 618.7899, 539.1788, 507.1549, 477.1614, 469.1360, 437.1502, 403.1254, 357.1203, 335.0217, 315.1093, 297.0993, 223.0613, 191.0349, 149.0235, 135.0443, 121.0285, 101.0233	-	neonuzhenide or isomer	Iridoids
41*	9.32	447.0951/449.1075[c]	2.137	12.5	$C_{21}H_{20}O_{11}$	447.0942, 327.0527, 297.0417, 285.0406, 256.0382, 227.0356, 199.0394, 175.0392, 151.0029, 133.0285, 107.0127	449.1044, 368.8729, 287.054, 269.0444, 241.0492, 203.0346, 153.0180	luteolin-7-*O*-glucosi de	Flavonoids
42	9.39	378.9783/-	3.034	12.5	$C_{15}H_8O_{10}S$	378.9777, 299.0202, 282.0156, 269.0092, 255.0302, 231.0301, 211.0397, 187.0396, 169.0289,	-	1,3,8,9-terahydroxyc oumestan	Coumarins

						151.0026, 135.0077, 110.1863			
43	9.63	623.2004/-	3.685	12.5	$C_{29}H_{34}O_{15}$	623.2004, 461.1662, 315.1111, 258.8888, 179.0344, 161.0236, 153.0544, 133.0284, 113.0232	-	verbascoside	Phenylethanols
44	9.64	477.1425/-	1.646	11.5	$C_{23}H_{26}O_{11}$	477.1410, 445.6834, 315.1091, 281.0675, 251.0568, 221.0457, 179.0344, 164.8359, 161.0237, 136.0474, 133.0285, 113.0235	-	3,4-dihydroxyphenethy l-(6'-caffeoyl)-β-D-g lucoside(or isomer)	Phenylethanols
45	10.02	515.1212/-	-4.043	5.5	$C_{25}H_{24}O_{12}$	515.1201, 382.3428, 353.0885, 335.0781, 273.0796, 203.0345, 191.0556, 179.0344, 173.0449, 161.0237, 135.0443, 111.0441	-	isochlorogenic acid C	Phenolic acids
46*	10.15	685.2349/704.2734[b]	2.681	11.5	$C_{31}H_{42}O_{17}$	523.1824, 453.1409, 421.1512, 299.1147, 271.9377, 223.0614, 179.053, 121.0284	475.1559, 369.1174, 295.0807, 225.0753, 165.0544, 151.0388	specnuezhenide	Iridoids
47	10.18	623.1996/-	3.781	12.5	$C_{29}H_{36}O_{15}$	623.2005, 461.1679, 315.1111, 288.6183, 221.4512, 200.8673, 179.0346, 161.0238, 151.0388, 133.0286, 113.0234	-	isomer of verbascoside	Phenylethanols
48	10.29	313.0365/-	2.792	12.5	$C_{16}H_{10}O_7$	313.0363, 300.0290, 285.0411, 272.0250, 270.0174, 245.0101, 242.0221, 218.0231, 216.0064, 211.0398, 198.0320, 186.0324, 165.0188, 148.0394, 139.0031, 122.0599, 109.0284	-	isomer of wedelolactone	Coumarins

49*	10.44	431.0984/433.1116[c]	1.531	12.5	$C_{21}H_{20}O_{10}$	431.0990, 395.9469, 311.0600, 268.0382, 240.0425, 151.0028, 107.0127	433.1132, 358.6413, 271.0595, 225.0546, 153.0180, 119.0492	apigenin-7-*O*-glucoside	Flavonoid
50	10.45	-/709.2302[a]	-1.947	10.5	$C_{31}H_{42}O_{17}$	-	709.2300, 602.4104, 547.1775, 515.1511, 473.1422, 323.1094, 165.0544	nuezhenide or isomer	Iridoids
51*	10.68	181.0505/-	-2.718	5.5	$C_9H_{10}O_4$	181.0501, 179.8413, 167.9042, 161.8754, 153.0186, 145.8615, 143.8643, 141.8674, 135.0078, 124.0159, 121.0283, 111.0077, 109.0284, 103.9191	-	ethyl protocatechuate	Phenolic acids
52	10.70	-/455.1515[a]	-1.764	5.5	$C_{19}H_{28}O_{11}$	-	455.1516, 394.1511, 311.2517, 293.0990, 261.0728, 219.0629, 165.0544,	benzyl Gentiobiosie or isomer	Iridoids
53*	10.77	-/302.1019[b]	-1.023	9.5	$C_{16}H_{12}O_5$	-	302.1020, 270.1121, 256.0963, 242.0803, 199.0625, 164.0704, 138.0550, 108.0445	acacetin	Flavonoid
54	10.86	515.1212/-	-4.043	5.5	$C_{25}H_{24}O_{12}$	515.1233, 468.9953, 353.0885, 299.0579, 203.0345, 191.0557, 179.0345, 173.0450, 155.0343, 135.0443, 127.0391, 111.0443	-	isochlorogenic acid B	Phenolic acids
55	11.18	364.9988/367.0113[c]	1.272	11.5	$C_{15}H_{10}O_9S$	364.9978, 285.0410, 255.0305, 199.0395, 133.0285, 123.0438, 107.0126	367.0113, 308.1122, 287.0545, 241.0492, 165.0545, 153.0180, 135.0439	kaempferol-3-sulfate	Flavonoids

56[*]	11.21	285.0412/-	2.065	11.5	$C_{15}H_{10}O_6$	285.0410, 283.0248, 267.0287, 255.0304, 243.0304, 229.0522, 211.0401, 199.0398, 185.0601, 175.0397, 159.0451, 151.0028, 147.0448, 133.0286, 121.0285, 107.0128	-	kaempferol	Flavonoids
57	11.32	364.9987/367.0118[c]	2.532	11.5	$C_{15}H_{10}O_9S$	364.9982, 331.9265, 285.0410, 255.0300, 243.0302, 217.0510, 199.0403, 175.0394, 151.0028, 133.0285, 107.0126	367.0113, 308.1122, 287.0545, 241.0492, 153.0180	luteolin-8-sulfate or isomer	Flavonoids
58	11.45	-/709.2304[a]	-2.031	10.5	$C_{31}H_{42}O_{17}$	-	709.2300, 619.1959, 547.1783, 323.1103, 121.0647	nuezhenide or isomer	Iridoids
59	11.52	539.1797/-	3.832	10.5	$C_{25}H_{32}O_{13}$	539.1791, 507,1508, 460.0672, 421.0894, 403.1241, 377.1249, 371.0972, 345.0987, 327.0885, 307.0830, 299.1144, 275.0929, 243.2337, 241.0723, 239.0561, 223.0612, 207.0292, 195.0661, 191.0343, 179.0555, 165.0188, 153.0550, 149.0237, 139.0391, 121.0285, 119.0490, 113.0234, 111.0077, 101.0233	-	oleuropein	Iridoids
60	11.60	-/709.2298[a]	-2.285	10.5	$C_{31}H_{42}O_{17}$	-	709.2298, 515.1514, 471.1590, 323.1087, 165.0542	nuezhenide or isomer	Iridoids

61	12.18	539.1791/-	2.589	10.5	$C_{25}H_{32}O_{13}$	539.1784, 461.3725, 403.1253, 377.1245, 371.1003, 345.0993, 315.1097, 307.0830, 275.0928, 241.0723, 223.0612, 209.0449, 191.0349, 165.0554, 139.0028, 121.0285	-	oleuropein or isomer	Iridoids
62	12.20	-/563.1725[a]	-1.389	9.5	$C_{25}H_{32}O_{13}$	-	563.1727, 401.1201, 327.0849, 203.0529, 165.0546, 137.0596	oleuropein or isomer	Iridoids
63*	12.43	269.0461/271.0601[c]	2.094	11.5	$C_{15}H_{10}O_5$	269.0461, 225.0555, 197.0610, 183.0444, 169.0653, 159.0444, 151.0027, 135.0443, 121.0287, 117.0335, 107.0128	271.0598, 225.0908, 178.0777, 153.0180, 119.0492	apigenin	Flavonoids
64*	12.87	285.0411/287.0546[c]	1.96	11.5	$C_{15}H_{10}O_6$	285.0410, 267.0306, 241.0514, 199.0397, 175.0395, 151.0029, 133.0285, 107.0127	287.0545, 269.0435, 241.0494, 213.0539, 161.0230, 153.0181, 135.0440	luteoline	Flavonoid
65*	12.91	1071.3562/1090.3973[b]	3.855	17.5	$C_{48}H_{64}O_{27}$	839.2621, 753.0600, 685.2361, 653.2118, 523.1832, 453.1414, 421.1520, 299.1142, 121.0285	695.2276, 611.3927, 369.1171, 225.0750, 165.0542	oleonuezhenide	Iridoids
66	13.01	285.0413/-	2.381	11.5	$C_{15}H_{10}O_6$	285.0411, 257.0453, 255.0305, 241.0500, 217.0504, 199.0401, 189.0548, 185.0604, 175.0393, 151.0031, 143.0505, 139.4444, 133.0287, 121.0283, 107.0129	-	isomer of luteoline	Flavonoids

67*	13.30	207.0663/-	-1.314	6.5	$C_{11}H_{12}O_4$	207.0660, 180.9408, 179.0344, 165.0190, 162.8383, 161.0237, 157.8623, 150.0312, 139.0391, 137.0234, 135.0442, 133.0284, 125.8722, 118.9411, 104.9246	-	ethyl caffeate	Phenolic acids
68*	13.31	313.0367/-	2.792	12.5	$C_{16}H_{10}O_7$	313,0363, 298.0125, 283.0252, 268.0019, 254.0223, 240.0067, 226.0273, 212.0114, 196.0158, 186.0321, 170.0369, 156.0195, 132.0193, 109.0269	-	wedelolactone	Coumarins
69*	13.50	-/593.1855[c]	-1.571	12.5	$C_{28}H_{32}O_{14}$	-	394.7499, 319.0821, 277.0704, 183.0649, 165.0544, 151.0388	acacetin-7-*O*-rutinos ide	Flavonoid
70*	13.55	519.1526/543.1473[a]	3.449	12.5	$C_{25}H_{28}O_{12}$	519.1526, 444.3269, 427.9951, 371.0998, 282.4253, 227.0558, 209.0454, 189.0553, 183.0659, 165.0550, 161.0601, 147.0443, 131.0492, 121.0648, 106.0412	543.1456, 455.3043, 315.0847, 251.0522, 225.0754, 139.0387	6'-O-trans-cinnamoy l 8-epikingisidic acid	Iridoids
71	14.52	269.0466/-	1.982	11.5	$C_{15}H_{10}O_5$	269.0461, 241.0513, 225.0558, 213.0556, 201.0555, 181.0648, 159.0445, 151.0029, 138.3531, 121.0285, 117.0335, 107.0128, 105.0334	-	isomer of apigenin	Flavonoids
72	14.67	701.3963/-	-2.736	2.5	$C_{32}H_{62}O_{11}S$	701.3946, 683.8875, 616.9285, 379.2826, 299.6938, 241.0026, 150.9699, 138.9697, 119.0487		eclalbasaponin X /eclalbasaponin IX	Triterpenoids
73	14.96	-/589.1885[a]	-2.295	10.5	$C_{27}H_{34}O_{13}$	-	589.1878, 529.1672, 365.1185,	fraxiresinol 1-*O*-gluc	others

							245.0807, 165.0544, 121.0649	oside or isomer	
74	14.97	519.1528/-	4.855	12.5	$C_{25}H_{28}O_{12}$	519.1523, 475.1615, 327.1087, 272.7083, 256.2371, 215.9377, 209.0459, 189.0554, 183.0658, 165.0553, 161.0601, 147.0444, 139.0026, 121.0649, 113.0233	-	6'-*O*-*cis*-cinnamoyl 8-epikingisidic acid or isomer	Iridoids
75*	15.19	-/556.2389[b]	-2.341	8.5	$C_{26}H_{34}O_{12}$	-	555.2753, 489.1753, 369.1176, 295.0803, 225.0753, 193.0487, 165.0544, 151.0387	ligulucidumoside A	Iridoid
76	15.28	909.3079/933.2999[a]	-3.496	7.5	$C_{42}H_{54}O_{22}$	909.3061, 839.2648, 685.2387, 540.2404, 523.1824, 513.9863, 479.1949, 453.1415, 421.1523, 385.1145, 308.1760, 299.1144, 281.1033, 241.0716, 223.0612, 197.0819, 153.0187, 121.0284, 101.0233	933.2971, 771.2441, 547.1767, 385.1258, 165.0542	6’-elenolylnicotiflorine	Iridoids
77	15.46	875.4145/-	1.834	9.5	$C_{42}H_{68}O_{17}S$	875.4120, 770.7531, 713.3587, 544.5336, 453.8809, 395.1407, 295.2355, 241.0025, 150.9700, 138.9698, 113.0235	-	ecliptasaponin Ⅵ or isomer	Triterpenoids
78	15.71	-/531.1829[a]	-1.549	9.5	$C_{25}H_{32}O_{11}$	-	531.1829, 499.1563, 458.0623, 396.1035, 352.1131, 322.1014, 292.3916, 266.5787, 218.0386, 189.0521, 167.0702, 129.0311	hirsutanonol 5-*O*-glucoside or isomer	others

79	15.91	795.4560/-	1.974	9.5	$C_{42}H_{68}O_{14}$	795.4552, 733.4496, 633.4016, 615.3884, 471.3484, 407.3329, 221.0669, 161.0448	-	ecliptasaponin I/IV/XV/C	Triterpenoids
80	16.21	701.3904/-	-1.149	11.5	$C_{36}H_{62}O_{11}S$	701.3898, 633.4021, 614.2345, 587.3940, 531.3417, 379.9699, 320.7715, 241.0029, 206.9143, 161.0448, 152.9949, 113.0233, 101.0233	-	isomer of eclalbasaponin X/ eclalbasaponin IX	Triterpenoids
81*	16.34	633.4028/-	1.458	8.5	$C_{36}H_{58}O_{9}$	633.4017, 491.1723, 357.2709, 231.8210, 186.8509, 161.0442, 143.0339, 129.0175, 113.0234, 101.0232	-	ecliptasaponin A/D	Triterpenoids
82	16.40	-/455.3501[c]	-1.234	7.5	$C_{30}H_{46}O_{3}$	-	455.3514, 437.3398, 409.3474, 391.3371, 315.7049, 265.1175, 247.1691, 229.1573, 219.1742, 191.1789, 187.1475, 161.1322, 147.1167	3-hydroxy-11-ursen-28,13-olide or isomer	others
83	16.48	713.3600/-	-3.019	4.5	$C_{32}H_{56}O_{17}$	713.3580, 619.7869, 551.2468, 512.4774, 475.8728, 374.6168, 307.6075, 355.3117, 241.0026, 222.9902, 200.5094, 180.9806, 150.9693, 138.9698, 115.2391, 113.0235	-	eclalbasaponin Ⅴ	Triterpenoids
84	16.52	701.3964/-	-2.123	2.5	$C_{32}H_{62}O_{11}S$	701.3950, 623.4960, 505.2403, 355.6421, 255.2341, 241.0027, 204.4177, 152.9952, 138.9899,	-	isomer of eclalbasaponin X/ eclalbasaponin IX	Triterpenoids

						116.9275			
85	16.81	487.3448/-	2.754	7.5	$C_{30}H_{48}O_5$	487.3442, 469.3336, 441.3398, 439.3214, 427.3235, 423.3279, 393.3175, 377.2842, 355.2637, 342.2047, 309.3189, 247.6271, 191.0540, 183.3537, 167.5094, 162.8387, 160.8410, 116.9273, 111.0805	-	tormentic acid	Triterpenoids
86	17.28	487.3447/-	2.508	7.5	$C_{30}H_{48}O_5$	487.3441, 471.3121, 469.3321, 451.3245, 439.3246, 427.3225, 423.3284, 409.3104, 405.5358, 385.2732, 331.2506, 313.2438, 201.1131, 173.1181, 171.1019, 144.3279, 116.9277, 115.9193, 111.0076	-	isomer of tormentic acid	Triterpenoids
87	17.42	269.0465/-	2.094	11.5	$C_{15}H_{10}O_6$	269.0461, 241.0510, 225.0557, 222.8195, 200.8802, 182.0367, 172.8858, 134.8937, 118.8986	-	isomer of apigenin	Flavonoids
88	18.14	633.3816/635.3942[c]	1.678	13.5	$C_{39}H_{54}O_7$	633.3807, 589.3902, 423.4019, 404.9825, 162.8384, 145.0286, 117.0035	565.5105, 435.3252, 409.3101, 389.3204, 259.1682, 201.1634, 147.0438	3-*O*-*cis*-*p*-*C*oumaroyltormenticacid/3-*O*-trans-p-coumaroyltormentic acid	Triterpenoids
89*	18.34	469.3340/471.3459[c]	1.954	8.5	$C_{30}H_{46}O_4$	469.3333, 423.3276, 393.2817, 159.4625	471.3463, 425.3410, 407.3280, 317.2103, 235.1691, 217.1584, 189.1635, 119.0856	16-hydroxy-3-oxoolean-12-en-28-oic acid	Triterpenoid

90*	18.67	471.3496/473.3614c	2.348	7.5	$C_{30}H_{48}O_4$	471.3491, 453.3375, 340.7722, 224.6396, 142.4072,	473.3626, 437.3397, 427.3564, 409.3457, 331.2617, 255.2107, 201.1635, 133.1012	echinocystic acid	Triterpenoid
91*	18.69	-/457.3665c	-1.556	6.5	$C_{30}H_{48}O_3$	-	457.3669, 439.3582, 410.3492, 303.2322, 221.1897, 189.1635, 135.1167, 121.1013	oleanolic acid	Triterpenoid
92	19.21	469.3341/-	1.89	8.5	$C_{30}H_{46}O_4$	469.3332, 425.3431, 407.3327, 391.3006, 375.6176, 137.0964, 116.9275	-	16-hydroxy-3-oxoolean-12-en-28-oic acid or isomer	Triterpenoids
93	19.65	617.3867/619.3993c	1.389	13.5	$C_{39}H_{54}O6$	617.3856, 497.3277, 453.3379, 352.4643, 282.9816, 226.8654, 163.0391, 145.0286, 121.0285 ,117.0335	482.2176, 437.3408, 261.1850, 203.1792, 147.0439	3β-O-trans-p-Coumaroylmaslinic acid or isomer /3β-O-cis-p-Coumaroylmaslinic acid or isomer	Triterpenoids
94	20.20	617.3868/619.3993c	1.097	13.5	$C_{39}H_{54}O6$	617.3854, 573.3968, 497.3262, 453.3377, 392.3151, 303.2892, 228.7577, 171.2528, 163.0393, 145.0286, 135.4542, 119.0492	549.6431, 409.3456, 203.1793, 147.0438	3β-O-trans-p-Coumaroylmaslinic acid or isomer /3β-O-cis-p-Coumaroylmaslinic acid or isomer	Triterpenoids
95	20.75	617.3870/-	1.778	13.5	$C_{39}H_{54}O6$	617.3859, 573.3967, 497.3277, 453.3396, 430.2642, 240.0895, 197.0809, 163.0395, 145.0287, 119.0492	-	3β-O-trans-p-Coumaroylmaslinic acid or isomer /3β-O-cis-p-Coumaroylmaslinic acid or isomer	Triterpenoids

96*	21.08	-/515.3720[c]	-2.117	7.5	$C_{32}H_{50}O_5$	-	485.3616, 469.3670, 437.3397, 373.2734, 301.2154, 255.2095, 215.1792, 189.1636, 119.0856	3*β*-O-acetylpomolic acid	Triterpenoid

[a]: precursor ions referring to $[M+Na]^+$;

[b]: precursor ions referring to $[M+NH_4]^+$;

[c]: precursor ions referring to $[M+H]^+$;

-: not detected or not available;

*: components identified by comparison with the reference compounds.

Table S3 Information of the 270 components failing to be characterized from EZP.

No.	t_R (min)	Parent ion (ESI-: MS^1/ESI+: MS^1)	Mass error (ppm)	RDB	Formula	ESI-: MS^2	ESI+: MS^2	Identification
1	0.67	-/203.0522[a]	-1.425	0.5	$C_6H_{12}O_6$	-	203.0253, 184.5853, 158.0806, 143.0186, 126.0548, 114.0549, 103.0390, 97.0285, 85.0284	unknown
2	0.70	-/266.1589[b]	-0.992	0.5	$C_{11}H_{20}O_6$	-	266.1595, 264.1251, 248.1125, 238.9845, 230.1021, 222.0763, 194.0815, 182.0812, 164.0705, 152.0703, 144.1018, 128.0706, 116.0707, 109.0284, 104.1073, 98.0604	unknown
3	0.70	-/144.1015[b]	-0.869	1.5	$C_7H_{10}O_2$	-	144.1018, 143.0814, 128.0197, 112.1965, 102.0552, 97.0763, 90.1954, 87.0445	unknown
4	0.78	209.0304/-	-1.894	1.5	$C_7H_{14}O_7$	209.0663, 207.0508, 194.3389, 191.0193, 173.0090, 159.0293, 147.0293, 135.0294, 129.0183, 122.0234, 115.0024, 101.0233, 89.0232, 87.0076	-	unknown
5	0.92	-/365.1044[a]	-0.144	1.5	$C_{12}H_{22}O_{11}$	-	347.0962, 275.0727, 255.8351, 208.9965, 203.0524, 190.9861, 185.0419, 172.9760, 143.0019, 128.9855, 98.9756	unknown
6	0.93	-/234.1331[b]	-0.680	1.5	$C_{10}H_{16}O_5$	-	232.1172, 216.1228, 214.1075, 206.9590, 198.1125, 186.0760, 162.9685, 156.1018, 144.1014, 127.0390, 118.0865, 109.0286, 97.0288	unknown

7	1.03	-/278.1227[b]	-2.691	2.5	$C_{11}H_{16}O_7$	-	278.1227, 262.1294, 260.1126, 244.1180, 242.1019, 232.1178, 224.0902, 216.1222, 214.1071, 200.0907, 196.0964, 180.1022, 166.0865, 154.0859, 142.0863, 130.0864, 128.0706, 116.0707, 112.0758, 100.0760	unknown
8	1.10	-/245.0627[a]	-0.710	1.5	$C_8H_{14}O_7$	-	245.0630, 233.8299, 228.1227, 226.1051, 211.5813, 198.1127, 184.0963, 168.0649, 158.9971, 138.0548, 127.0390, 120.0808, 112.0508, 109.0284, 97.0288	unknown
9	1.42	353.0741/-	-0.281	4.5	$C_{12}H_{18}O_{12}$	353.0724, 317.2755, 268.7864, 243.3997, 202.3602, 191.0556, 181.0709, 179.0557, 173.0087, 169.8493, 164,8348, 154.9976, 129.0182, 122.8934, 120.9680, 119.0336, 111.0077, 103.9190, 101.0235	-	unknown
10	1.44	-/212.0526[c]	0.619	1.0	$C_6H_{11}O_8$	-	212.0528, 210.1123, 194.0806, 192.1017, 179.0941, 168.0651, 152.0704, 138.0914, 136.0393, 124.0758, 120.0808, 113.9636, 108.0447, 95.0494, 87.0445	unknown
11	1.44	-/231.0836[a]	-0.473	0.5	$C_8H_{16}O_6$	-	231.0838, 229.1544, 213.1226, 194.0810, 186.0765, 170.0809, 151.0620, 142.0861, 131.9742, 124.0753, 114.0550, 109.0286, 97.0289	unknown

12	1.55	-/159.0652[c]	-1.354	2.5	$C_7H_{10}O_4$	-	159.0650, 158.0923, 154.9901, 148.9768, 141.9587, 135.9446, 131.9742, 128.9507, 125.9606, 117.9594, 113.9639, 112.0872, 107.9504, 104.0710, 101.0600, 97.9691, 95.0493, 90.9481, 87.0446, 86.0605, 85.0287	unknown
13	1.58	-/300.1434[b]	-1.412	4.5	$C_{14}H_{18}O_6$	-	300.1437, 282.1320, 259.0297, 224.0921, 210.1125, 193.0019, 182.0811, 167.0708, 150.0551, 138.0912, 127.0389, 120.0809, 109.0286, 97.0287	unknown
14	1.93	-/294.1542[a]	0.617	1.5	$C_{12}H_{20}O_7$	-	294.1549, 278.1418, 276.1439, 264.1228, 258.1333, 248.1490, 230.1385, 226.8935, 212.1281, 208.8831, 194.1173, 170.1175, 161.0684, 152.0566, 144.1018, 132.1019, 116.1071, 109.0285, 98.0968, 88.0397	unknown
15	2.05	-/276.1437[b]	0.131	2.5	$C_{12}H_{18}O_6$	-	276.1442, 258.1334, 248.1493, 244.0598, 230.1384, 226.8933, 215.9015, 212.1279, 208.8828, 197.8909, 194.1176, 180.8876, 168.1017, 161.0677147.0140, 144.1019, 132.1019, 127.0391, 115.0393, 112.0396, 109.0285, 97.0289, 88.0398	unknown
16	2.09	-/300.1436[b]	-1.912	4.5	$C_{14}H_{18}O_6$	-	300.1436, 289.4321, 282.1328, 264.1240, 240.1230, 226.8934, 208.8834, 180.1012, 167.0700, 152.0706, 138.0912, 127.0389, 120.0809, 110.0603, 97.0286	unknown

17	2.16	-/231.0840[a]	0.782	0.5	$C_8H_{16}O_6$	-	231.0841, 229.1545, 212.0918, 194.0814, 182.0810, 164.0709, 152.1338, 142.0861, 129.0544, 127.0390, 124.0757, 111.0442, 106.0654, 98.0602, 88.0399, 86.0605	unknown
18	2.28	-/174.1488[b]	-1.237	0.5	$C_9H_{16}O_2$	-	174.1486, 172.1326, 167.9512, 154.9898, 149.9404, 144.9656, 133.9293, 128.0705, 113.9638, 107.9510, 101.0236, 98.9754, 92.8995, 90.9036, 87.0445, 75.0445	unknown
19	2.32	-/260.1602[a]	2.114	-1.0	$C_{11}H_{25}O_5$	-	260.1600, 242.1496, 237.9100, 224.1389, 216.1209, 205.0966, 188.0513, 177.1025, 163.0867, 147.0913, 127.0389, 111.0441, 99.0444, 93.0704	unknown
20	2.58	-/224.1278[b]	-1.740	4.5	$C_{12}H_{14}O_3$	-	224.1277, 222.1123, 200.9722, 188.3475, 177.9564, 164.0706, 155.9743, 138.0912, 132.9584, 124.0393, 120.0808, 113.9640, 101.9499, 88.0477	unknown
21	2.60	393.1419/-	2.939	4.5	$C_{16}H_{26}O_{11}$	393.1414, 375.1307, 363.1304, 349.1514, 331.1407, 284.9261, 240.9858, 213.0764, 195.0662, 183.0655, 161.0453, 151.0757, 139.0755, 125.0962, 113.0234, 101.0233	-	unknown
22	2.70	-/314.1226[b]	-0.154	5.5	$C_{14}H_{16}O_7$	-	314.1234, 296.1494, 269.1121, 249.0288, 224.0909, 211.1081, 192.0654, 167.0700, 152.0705, 147.0327, 127.0390, 120.0445, 109.0284, 95.0495	unknown

23	2.78	-/417.1358[a]	-1.061	3.5	$C_{16}H_{26}O_{11}$	-	417.1363, 400.1647, 284.0944, 255.0842, 237.0731, 223.0606, 203.0524, 195.0639, 164.0706, 127.0388, 111.0442, 97.0288	unknown
24	2.93	-/284.1490[b]	-2.215	4.5	$C_{14}H_{18}O_5$	-	284.1486, 266.1377, 237.0275, 219.0168, 206.0324, 192.1016, 183.0177, 166.0861, 153.0404, 136.0616, 127.0389, 122.0965, 114.0548, 107.0732, 85.0289	unknown
25	3.09	-/313.0889[a]	-1.657	3.5	$C_{12}H_{18}O_8$	-	294.1330, 269.1120, 258.1121, 225.0750, 220.0965, 211.1075, 198.1125, 194.1180, 174.0907, 167.0700, 160.0753, 150.0546, 135.0655, 123.0441, 120.0809, 111.0443, 101.0236	unknown
26	3.18	329.0893/-	2.293	6.5	$C_{14}H_{18}O_9$	329.0886, 295.0819, 261.3461, 209.0462, 191.0350, 179.0341, 167.0345, 164.0110, 151.0393, 139.0391, 135.0442, 123.0442, 113.0234, 109.0284, 101.0230	-	unknown
27	3.25	393.1421/-	3.105	4.5	$C_{16}H_{25}O_{11}$	393.1414, 375.1303, 329.0898, 290.3916, 213.0764, 183.0665, 151.0757, 139.0756, 113.0233, 101.0234	-	unknown
28	3.31	-/242.1495[a]	2.992	0.0	$C_{11}H_{23}O_4$	-	242.1496, 240.1229, 224.1390, 215.0967, 201.9743, 181.0490, 168.0656, 148.9774, 137.0596, 121.0649, 109.0286, 97.0288	unknown
29	3.56	169.0868/-	-3.475	3.5	$C_9H_{14}O_3$	169.0864, 167.0346, 157.8623, 151.0026, 142.9458, 137.0235, 132.8670, 128.0339, 126.8801, 123.0805, 121.0651, 114.9506, 109.0285, 103.9191	-	unknown

30	4.00	315.1101/339.1050[a]	1.584	5.5	$C_{14}H_{20}O_8$	315.1090, 305.0864, 277.9211, 224.9408, 211.7664, 193.7211, 179.0550, 162.8384, 153.0550, 150.0314, 135.0442, 123.0442, 119.0339, 109.0284, 101.0233	339.1045,235.0191,178.0860	unknown
31	4.06	-/339.1045[a]	-1.530	4.5	$C_{14}H_{20}O_8$	-	339.1045, 235.0191, 178.0860, 120.0809	unknown
32	4.07	349.1517/-	-0.785	2.5	$C_{16}H_{30}O_4S_2$	349.1510, 315.1094, 294.9561, 223.3155, 187.0969, 169.0863, 153.0550, 135.0443, 123.0442, 113.0234, 101.0233	-	unknown
33	4.09	-/373.1462[a]	-1.296	2.5	$C_{15}H_{26}O_9$	-	373.1464,229.0005,193.0833, 137.0593,74.0970	unknown
34	4.09	-/171.1013[c]	-0.823	2.5	$C_9H_{14}O_3$	-	171.1014, 148.9768, 128.9508, 111.0807, 93.0702	unknown
35	4.14	389.1106/413.1042[a]	2.609	6.5	$C_{16}H_{22}O_{11}$	389.1100, 335.3665, 227.0560, 209.0451, 183.0658, 179.0553, 165.0551, 161.0443, 139.0753, 131.0341, 121.0649, 113.0233, 101.0234	413.1047, 394.1861, 280.1033, 251.0522, 233.0421, 120.0806	unknown
36	4.14	313.0943/-	2.361	6.5	$C_{14}H_{18}O_8$	313.0936, 193.0496, 180.9958, 179.0558, 160.8417, 159.8588, 153.0549, 151.0393, 143.8644, 137.0230, 135.0442, 123.0441, 113.0234, 109.0286, 101.0232	-	unknown
37	4.21	213.0408/-	0.229	5.5	$C_9H_{10}O_6$	213,0405, 195.0301, 186.8974, 181.0498, 169.0502, 157.8627, 151.0394, 141.0184, 127.0654, 125.0599, 122.8932, 114.0551, 107.0492	-	unknown
38	4.27	-/371.1307[a]	-1.464	3.5	$C_{15}H_{24}O_9$	-	371.1307, 354.1198, 238.0924, 192.0873, 114.0914	unknown

39	4.32	219.0514/-	-0.347	3.5	$C_8H_{11}O_7$	219.0510, 216.8433, 184.8757, 173.0084, 159.0289, 157.0499, 154.9968, 129.0548, 122.2007, 113.0596, 111.0077	-	unknown
40	4.43	215.0564/-	-0.890	4.5	$C_9H_{12}O_6$	215.0559, 197.0452, 186.8969, 171.0657, 168.8857, 159.8592, 157.8623, 153.0551, 144.0081, 133.0287, 127.0755, 122.8932, 117.8642, 111.0078, 109.0648, 100.0755	-	unknown
41	4.64	461.1685/485.1627[a]	2.690	6.5	$C_{20}H_{30}O_{12}$	461.1677, 315.1096, 297.0978, 221.0656, 177.0544, 161.0447, 143.0339, 135.0443, 123.0438, 113.0234	485.1623, 399.1042, 164.0706	unknown
42	4.72	347.1361/-	1.972	4.5	$C_{15}H_{24}O_9$	347.1354, 329.9627, 323.9091, 313.0932, 311.9503, 309.9107, 297.2379, 288.0899, 277.0930, 257.1041, 254.9524, 223.5259, 210.6245, 198.9018, 197.0815, 185.0815, 184.0696, 181.0713, 169.0863, 168.0413, 165.0552, 163.0609, 155.0709, 151.0394, 143.0344, 141.7569, 139.0756, 131.0342, 123.0441, 113.0232, 101.0233	-	unknown
43	4.85	-/323.1093[a]	-1.189	4.5	$C_{14}H_{20}O_7$	-	323.1097, 275.7556, 204.4594, 121.0648	unknown
44	4.96	553.1798/-	1.685	6.5	$C_{22}H_{34}O_{16}$	553.1783, 389.1116, 371.0986, 338.4529, 269.8676, 231.8200, 209.0457, 191.0345, 181.0713, 165.0555, 149.0240, 121.0285, 101.0233	-	unknown
45	4.98	-/426.1360[a]	1.955	1.0	$C_{14}H_{27}O_{13}$	-	426.1352, 363.5099, 304.1179, 264.0838, 232.0578, 164.0342	unknown

46	5.12	-/431.1516[a]	-1.931	3.5	$C_{17}H_{28}O_{11}$	-	431.1516, 409.9910, 269.0989, 251.0889, 203.0519	unknown
47	5.21	-/457.0940[a]	-2.498	6.5	$C_{17}H_{22}O_{13}$	-	457.0941, 322.6229, 295.0418, 263.0158, 185.0415	unknown
48	5.27	373.0792/-	-0.495	7.5	$C_{15}H_{18}O_{11}$	373.0775, 329.0887, 301.0934, 266.6675, 209.0437, 193.0140, 181.0502, 167.0343, 152.0107, 139.0391, 124.0155, 113.0231	-	unknown
49	5.27	443.1943/-	2.188	6.5	$C_{21}H_{32}O_{10}$	443.1932, 368.6536, 297.3500, 238.2006, 219.1387, 189.1284, 160.8412, 119.0338, 113.0233, 101.0233	-	unknown
50	5.32	-/216.1223[a]	-0.437	2.5	$C_{10}H_{14}O_4$	-	216.1229, 180.1014, 155.0701, 133.9290, 113.0599, 104.0709	unknown
51	5.44	227.0567/-	0.523	5.5	$C_{10}H_{12}O_6$	227.0562, 226.1236, 224.1094, 211.0992, 209.0452, 198.0920, 191.0342, 183.0679, 167.0344, 165.0551, 153.0547, 139.0392, 127.0389, 123.0441, 119.0492, 111.0441	-	unknown
52	5.75	-/355.1356[a]	-1.686	3.5	$C_{15}H_{24}O_8$	-	355.1357, 316.0238, 264.8702, 163.0388	unknown
53	5.75	-/589.1727[a]	-1.164	6.5	$C_{23}H_{34}O_{16}$	-	589.1732, 528.9596, 427.1205, 395.0944, 265.0688, 165.0541	unknown
54	5.78	-/369.1169[a]	-1.743	4.5	$C_{15}H_{22}O_9$	-	369.1150, 352.1157, 267.0866, 225.0753, 165.0546, 151.0388	unknown
55	5.92	-/171.1014[c]	-0.239	2.5	$C_9H_{14}O_3$	-	171.1015, 148.9769, 128.9507, 111.0806, 105.0699, 89.9403	unknown
56	6.00	-/489.1203[a]	-1.649	5.5	$C_{18}H_{26}O_{14}$	-	489.1207, 457.0956, 327.0681, 295.0424, 209.0416	unknown

57	6.07	-/450.1747[c]	2.671	5.0	$C_{19}H_{29}O_{12}$	-	450.1744, 288.1225, 270.1120, 209.0831, 150.0548, 85.0289	unknown
58	6.20	179.0349/-	-2.413	6.5	$C_9H_8O_4$	179.0345, 177.8448, 161.8751, 152.9168, 143.8643, 136.0476, 135.0443, 133.0287, 124.8909, 119.0493, 107.0491	-	unknown
59	6.30	-/365.1202[a]	-1.393	5.5	$C_{16}H_{22}O_8$	-	365.1202, 331.7239, 165.0785, 121.0648	unknown
60	6.60	-/243.1224[c]	-2.921	3.5	$C_{12}H_{18}O_5$	-	243.1220, 211.0962, 183.1009, 169.0857, 139.0752, 109.0650	unknown
61	6.60	-/445.1671[a]	-1.646	3.5	$C_{18}H_{30}O_{11}$	-	445.1673, 372.7794, 267.1179, 201.0367	unknown
62	6.70	-/413.1408[a]	-1.327	4.5	$C_{17}H_{26}O_{10}$	-	413.1413, 233.1741, 165.0532	unknown
63	6.87	553.2084/-	-0.941	14.5	$C_{30}H_{34}O_{10}$	553.2074, 517.2306, 293.0884, 233.0663, 205.1235, 191.0563, 179.3073, 153.0908, 149.0445, 113.0235, 101.0230	-	unknown
64	7.02	-/490.2273[b]	-0.974	5.5	$C_{22}H_{32}O_{11}$	-	490.2278, 431.1544, 328.1764, 251.0910, 183.0650, 165.0545, 123.0442	unknown
65	7.13	-/337.2228[c]	1.987	0.5	$C_{16}H_{32}O_7$	-	337.2227, 293.2340, 224.1391, 197.1395, 126.1026, 114.0914, 84.0561	unknown
66	7.15	593.1539/-	2.152	13.5	$C_{27}H_{30}O_{15}$	593.1525, 503.1171, 473.1096, 413.0903, 395.0763, 383.0799, 365.0666, 353.0676, 337.0739, 325.0722, 311.0566, 297.0773, 283.0609, 268.0746, 233.0451, 191.0339, 161.0233, 135.0449, 117.0336	-	unknown

67	7.28	-/224.1391[a]	2.674	1.0	$C_{11}H_{21}O_3$	-	224.1389, 204.1382, 177.9561, 165.0545, 152.0703, 139.0388, 113.9639, 101.9501	unknown
68	7.39	-/292.1903[b]	-2.259	5.5	$C_{17}H_{22}O_3$	-	292.1901, 230.0805, 184.1331, 172.1329, 121.0648, 93.0702	unknown
69	7.58	-/322.2007[b]	-2.529	5.5	$C_{18}H_{24}O_4$	-	322.2005, 277.1895, 230.1531, 184.1325, 138.0911, 121.0648, 110.0966	unknown
70	7.73	-/485.1261[a]	-1.962	6.5	$C_{19}H_{26}O_{13}$	-	485.1256, 368.1511, 323.0733, 291.0469, 261.0720	unknown
71	7.73	-/207.1380[c]	-1.045	4.5	$C_{13}H_{18}O_2$	-	207.1377, 149.0961, 123.0805, 95.0859, 84.9602	unknown
72	7.81	-/365.1202[a]	-1.147	5.5	$C_{16}H_{22}O_8$	-	365.1203, 334.2791, 289.4790, 164.0701, 121.0645	unknown
73	7.82	281.1042/-	-0.432	6.5	$C_{14}H_{18}O_6$	281.1029, 231.4816, 219.8446, 191.0348, 161.0450, 147.0289, 137.0398, 129.0181, 119.0493, 113.0233, 103.9193	-	unknown
74	8.03	545.0624/-	-1.791	7.5	$C_{17}H_{22}O_{20}$	545.0622, 502.0647, 465.1059, 460.7359, 425.4771, 303.0522, 285.0413, 247.3224, 241.0025, 222.9916, 207.0295, 193.0139, 168.9805, 165.0186, 150.9696, 138.9698, 113.0232, 109.0284	-	unknown
75	8.09	-/439.1565[a]	-1.658	5.5	$C_{19}H_{28}O_{10}$	-	439.1567, 307.1146, 233.9962, 121.0649	unknown
76	8.34	-/236.1646[b]	-1.378	4.5	$C_{14}H_{18}O_2$	-	236.1642, 219.0651, 165.0783, 135.0805, 122.0965, 107.0733	unknown

77	8.58	259.0623/-	1.750	9.5	$C_{14}H_{12}O_5$	259.0616, 241.0504, 223.8392, 221.8425, 219.8453, 213.0554, 201.0552, 195.0447, 187.0399, 175.0393, 173.0602, 159.0444, 149.0234, 137.0599, 133.0284, 131.0491, 109.0286	-	unknown
78	8.83	-/459.1833[a]	-1.248	3.5	$C_{19}H_{32}O_{11}$	-	459.1831, 429.1719, 281.1360, 164.0699	unknown
79	8.92	527.0527/-	1.648	8.5	$C_{17}H_{20}O_{19}$	527.0535, 288.2475, 285.0410, 255.0313, 241.0024, 217.0507, 199.0391, 175.0391, 175.0396, 151.0030, 133.0287	-	unknown
80	9.00	195.0663/-	0.348	5.5	$C_{10}H_{12}O_4$	195.0664, 193.8149, 165.0552, 160.8415, 158.8461, 151.0757, 137.2468, 123.0442, 121.0647, 103.9191	-	unknown
81	9.27	-/593.1470[a]	-1.115	10.5	$C_{25}H_{30}O_{15}$	-	593.1470, 517.8396, 431.0943, 399.0680, 263.0159, 137.0596	unknown
82	9.34	-/449.1071[a]	-2.299	8.5	$C_{19}H_{22}O_{11}$	-	449.1044, 368.8729, 287.0545, 241.0492, 153.0180	unknown
83	9.43	297.0052/-	1.900	13.5	$C_{15}H_6O_7$	297.0046, 289.2991, 271.0161, 269.0097, 253.0147, 241.0148, 225.0194, 213.0190, 210.0318, 197.0237, 185.0237, 169.0287, 157.0290, 141.0336, 135.0078, 129.0336, 117.1785	-	unknown
84	9.62	-/209.1534[c]	-1.943	3.5	$C_{13}H_{20}O_2$	-	209.1532, 165.1271, 143.9966, 125.0233	unknown
85	9.68	481.1737/-	2.359	9.5	$C_{23}H_{30}O_{11}$	481.1727, 347.1365, 299.1147, 250.7534, 181.0502, 161.0238, 137.0597, 121.0285, 111.0077	-	unknown

86	9.74	-/407.1310[a]	-1.801	6.5	$C_{18}H_{24}O_9$	-	407.1305, 355.1071, 244.0965, 164.0705	unknown
87	10.36	321.0992/-	-0.050	8.5	$C_{16}H_{18}O_7$	321.0980, 173.0450, 162.9986, 155.0342, 147.0443, 137.0235, 111.0441	-	unknown
88	10.38	-/311.1458[a]	-2.441	2.5	$C_{14}H_{24}O_6$	-	311.1458, 293.1367, 249.1455, 170.0923	unknown
89	10.64	539.1792/-	4.277	10.5	$C_{25}H_{32}O_{13}$	539.1793, 396.8874, 377.1248, 359.1150, 345.0993, 291.0879, 275.0930, 243.1079, 239.0557, 207.0293, 179.0340, 171.0294, 137.0599, 127.0390, 111.0076, 101.0233	-	unknown
90	10.66	-/658.2482[c]	2.634	10.0	$C_{30}H_{41}O_{16}$	-	685.2485, 496.1957, 464.1697, 424.6213, 306.7371, 193.0491, 165.0544, 121.0648	unknown
91	10.80	-/236.2006[b]	-2.036	3.5	$C_{15}H_{22}O$	-	236.2004, 190.1587, 166.1224, 135.1044, 120.0808	unknown
92	11.07	321.0995/-	2.317	8.5	$C_{16}H_{18}O_7$	321.0987, 310.2601, 265.3546, 173.0451, 155.0342, 147.0444, 143.0341, 129.0183, 115.0389, 111.0441	-	unknown
93	11.32	379.1049/-	-0.252	9.5	$C_{18}H_{20}O_9$	379.1034, 348.9791, 301.0363, 283.0282, 255.0302, 243.0509, 199.0609, 190.6661, 167.0349, 155.0706, 149.0235, 135.0446, 123.0441, 108.0203	-	unknown
94	11.62	581.1899/-	-2.338	11.5	$C_{27}H_{34}O_{14}$	581.1862, 417.1402, 375.1454, 366.2139, 291.0878, 273.1136, 237.0771, 193.0868, 171.0292, 135.0441, 127.0391, 111.0077, 101.0233	-	unknown

95	11.70	-/506.2011[c]	1.655	6.0	$C_{22}H_{33}O_{13}$	-	506.2002, 366.1184, 302.1379, 225.0751, 165.0543, 139.0387	unknown
96	11.79	243.1245/-	1.246	3.5	$C_{12}H_{20}O_5$	243.1241, 227.1191, 225.1134, 222.8147, 207.1025, 199.1337, 197.1337, 181.1230, 163.1122, 155.1431, 142.0265, 134.8937, 125.0960, 110.0363, 103.9193	-	unknown
97	11.81	-/751.2403[a]	-1.844	11.5	$C_{33}H_{44}O_{18}$	-	751.2406, 589.1868, 547.1781, 515.1509, 323.1093, 165.0544	unknown
98	11.89	-/209.1537[c]	-1.369	3.5	$C_{13}H_{20}O_2$	-	209.1533, 165.1272, 143.9967, 102.9705, 84.9602	unknown
99	12.04	-/519.1826[a]	-1.700	8.5	$C_{24}H_{32}O_{11}$	-	519.1828, 404.3011, 237.0732, 133.1009	unknown
100	12.75	-/579.2047[a]	-0.280	8.5	$C_{26}H_{36}O_{13}$	-	579.2047, 547.1783, 445.1093, 305.0986, 165.0543	unknown
101	13.08	523.1841/-	-1.222	10.5	$C_{25}H_{32}O_{12}$	523.1815, 479.1956, 453.1414, 447.1669, 421.1517, 299.1143, 291.0878, 259.0978, 223.0615, 197.0817, 179.0552, 160.8415, 153.0186, 137.0598, 119.0492, 101.0233	-	unknown
102	13.10	-/669.2639[b]	-1.889	2.0	$C_{24}H_{43}O_{20}$	-	669.2673, 531.1967, 429.1667, 369.1440, 337.1176, 279.1122, 121.0649	unknown
103	13.34	347.0421/-	2.146	11.5	$C_{16}H_{12}O_9$	347.0416, 330.7712, 303.0515, 288.0285, 269.0460, 259.0615, 247.0270, 244.0378, 229.0145, 205.0140, 192.0059, 165.0187, 152.0105, 137.0235, 124.0158	-	unknown

104	13.57	-/543.1464[a]	-1.376	11.5	$C_{25}H_{28}O_{12}$	-	543.1465, 455.3043, 315.0847, 251.0522, 139.0387	unknown
105	13.73	-/793.2512[b]	2.745	7.0	$C_{29}H_{43}O_{24}$	-	793.2504, 722.6218, 631.1970, 589.1888, 557.1633, 365.1213, 165.0543	unknown
106	13.84	-/308.2214[b]	-1.688	4.5	$C_{18}H_{26}O_{3}$	-	308.2215, 290.2109, 262.2160, 220.9056, 179.1303, 122.0601	unknown
107	14.04	-/561.1935[a]	-1.475	9.5	$C_{26}H_{34}O_{12}$	-	561.1934, 529.1701, 434.1388, 305.1001, 165.0545	unknown
108	14.25	327.2188/-	2.086	3.5	$C_{18}H_{32}O_{5}$	327.2184, 309.2087, 291.1973, 273.1865, 261.1881, 203.1184, 201.1130, 183.1025, 171.1022, 155.1069, 137.0960, 127.1121, 107.0855	-	unknown
109	14.27	-/1095.3494[b]	1.196	11.0	$C_{42}H_{61}O_{32}$	-	1095.3497,933.2969,739.2186,547.1763, 165.0544	unknown
110	14.43	-/933.2983[b]	1.632	10.0	$C_{36}H_{51}O_{27}$	-	933.2971, 771.2464, 721.2011, 547.1793, 413.1219, 218.1168, 165.0544	unknown
111	14.77	-/235.1690[c]	-1.899	4.5	$C_{15}H_{22}O_{2}$	-	235.1688, 217.1586, 189.1271, 159.1165, 121.1012, 111.0806	unknown
112	14.81	-/353.2290[a]	-1.600	1.5	$C_{18}H_{34}O_{5}$	-	353.2293, 294.1128, 243.1091, 164.0716	unknown
113	14.85	-/277.2156[c]	-1.611	4.5	$C_{18}H_{28}O_{2}$	-	277.2158, 259.2053, 195.1378, 149.0230, 135.1167, 121.1012, 93.0703	unknown

114	15.04	-/275.2000[c]	-1.550	5.5	$C_{18}H_{26}O_2$	-	275.2001, 257.1892, 239.1791, 229.1953, 215.9002, 197.1319, 173.1323, 159.1164, 147.1165, 135.1170, 133.1011, 121.1013, 119.0856, 107.0858, 105.0701, 95.0860, 93.0703, 91.0546	unknown
115	15.08	-/503.1880[a]	-1.606	8.5	$C_{24}H_{32}O_{10}$	-	503.1880, 489.2202, 471.2119, 341.1366, 323.1254, 300.1228, 271.0597, 203.0524, 175.1781, 164.0700, 151.0397, 119.0856	unknown
116	15.14	-/575.2088[a]	-1.056	9.5	$C_{27}H_{36}O_{12}$	-	575.2093, 529.1740, 503.1516, 471,1250, 453.2065, 323.1098, 293.1007, 247.0567, 229.0475, 165.0542, 121.0258	unknown
117	15.33	-/561.1929[a]	-1.368	9.5	$C_{26}H_{34}O_{12}$	-	561.1935, 492.7903, 468.4950, 388.0598, 340.0244, 265.9855, 247.0569, 208.9881, 181.0854, 167.0701, 151.0388, 121.0644	unknown
118	15.35	345.2292/369.2238[a]	2.225	2.5	$C_{18}H_{34}O_6$	345.2290, 327.2185, 309.2078, 291.1974, 273.1845, 265.2182, 247.2072, 201.1129, 183.1024, 171.1020, 155.1073, 139.1122, 125.0963	369.2242, 356.1115, 338.0995, 327.1334, 311.1022, 297.0878, 294.1113, 267.1130, 252.0883, 225.0770, 205.0885, 181.0868, 169.0369, 165.0544, 153.0545, 139.0387, 121.0647	unknown
119	15.37	-/437.3403[c]	-1.343	8.5	$C_{30}H_{44}O_2$	-	437.3408, 419.3316, 405.1455, 391.3350, 377.1486, 369.1445, 343.1447, 311.3377, 287.2011, 255.2095, 247.1687, 215.1492, 201.1636, 189.1634, 175.1477, 165.0545, 159.1166, 145.1012, 133.1011, 119.0856, 107.0857, 95.0859	unknown

120	15.42	331.2504/355.2446[a]	2.574	1.5	$C_{18}H_{36}O_5$	331.2498, 327.2173, 313.2390, 295.2281, 271.0971, 253.0509, 201.1134, 187.1334, 171.1019, 157.1227, 143.1070, 127.1118, 123.0800	355.2448, 266.8707, 248.4823, 227.1405, 211.5492, 182.2383, 164.0699, 151.0390, 95.0495	unknown
121	15.44	-/297.2418[c]	-2.831	2.5	$C_{18}H_{32}O_3$	-	297.2416, 279.2314, 261.2208, 246.5650, 243.2110, 223.1701, 209.1536, 187.1483, 173.1325, 161.1321, 137.1324, 123.1168, 121.1012, 109.1014, 97.0651, 95.0859, 93.0702, 81.0704	unknown
122	15.46	377.1256/401.1207[a]	1.987	9.5	$C_{19}H_{22}O_8$	377.1249, 345.1005, 327.0883, 307.0828, 301.1092, 275.0568, 241.0726, 231.1034, 209.0448, 197.0820, 171.0290, 165.0550, 149.0236, 139.0027, 127.0391, 111.0077, 101.0233	401.1198,327.1324,265.0673,164.0703	unknown
123	15.48	-/351.2138[a]	-1.894	2.5	$C_{18}H_{32}O_5$	-	351.2135, 335.1276, 319.0821, 277.0700, 259.0600, 245.0801, 225.1102, 199.0385, 185.0603, 177.0534, 165.0544, 151.0381, 137.0595, 109.0286	unknown
124	15.56	-/246.2424[b]	-1.242	-0.5	$C_{14}H_{28}O_2$	-	246.2424, 2228.2320, 202.2161, 194.1064, 143.0843, 119.0857, 106.0865, 89.0794, 88.0761, 85.1015	unknown
125	15.60	-/277.2160[c]	-1.936	4.5	$C_{18}H_{28}O_2$	-	277.2157, 259.2049, 241.1945, 221.1532, 207.1376, 179.1432, 171.1167, 163.1119, 149.1324, 135.1167, 131.0856, 121.1012, 109.1012, 107.0858, 99.0807, 95.0859	unknown

126	15.60	-/529.1670[a]	-1.744	10.5	$C_{25}H_{30}O_{11}$	-	529.1671, 453.4401, 401.1491, 368.1440, 322.1019, 298.1189, 270.1149, 225.0748, 187.0365, 165.0544, 151.0387, 121.0649	unknown
127	15.62	401.0891/-	1.508	12.5	$C_{20}H_{18}O_9$	401.0884, 357.0619, 313.0723, 282.0173, 258.6857, 225.0557, 209.0445, 181.0653, 177.0918, 160.8414, 126.4773, 121.0284, 116.9271, 114.1606	-	unknown
128	15.63	-/353.2295[a]	-2.025	1.5	$C_{18}H_{34}O_5$	-	353.2291, 333.9936, 279.6403, 250.8285, 223.4868, 212.7228, 187.7787, 164.0705, 137.0593, 125.0234	unknown
129	15.64	-/290.2685[b]	-2.275	-0.5	$C_{16}H_{32}O_3$	-	290.2683, 273.1849, 230.2387, 228.1318, 184.2050, 164.0703, 145.1010, 133.1009, 119.0853, 105.0700, 102.0916, 93.0703	unknown
130	15.64	-/575.2089[a]	-1.369	9.5	$C_{27}H_{36}O_{12}$	-	575.2091, 543.1817, 497.1425, 477.1367, 447.1597, 413.1591, 322.1029, 293.1004, 261.0741, 233.0782, 201.0515, 165.0544	unknown
131	15.74	-/235.2054[c]	-2.645	3.5	$C_{16}H_{26}O$	-	235.2050, 233.1534, 217.1937, 199.1472, 191.1792, 177.1632, 175.1477, 165.0783, 161.1322, 151.1476, 147.1166, 137.1321, 133.1010, 123.1169, 121.1012, 119.0856,107.0857, 105.0701, 97.0651	unknown
132	15.93	-/917.3013[b]	1.568	10.0	$C_{36}H_{51}O_{26}$	-	917.3021, 755.2501, 723.2251, 681.1128, 653.2188, 547.1790, 513.1729, 459.1672, 397.1252, 347.1108, 305.0978, 231.0627	unknown

133	16.11	-/309.2054[a]	1.615	1.5	$C_{16}H_{30}O_4$	-	309.2041, 299.8710, 291.1946, 273.1841, 266.9368, 255.1735, 243.9218, 238.9166, 223.1310, 210.9215, 202.8952, 189.1121, 174.9000, 161.0956, 147.0801, 125.0963, 119.0855	unknown
134	16.18	-/394.1754[a]	1.268	9.0	$C_{22}H_{27}O_5$	-	394.1756, 379.1523, 362.1493, 334.1540, 219.1298, 304.1439, 274.0973, 257.1287, 242.1045, 215.0812	unknown
135	16.26	-/573.1934[a]	-1.339	10.5	$C_{27}H_{34}O_{12}$	-	573.1935, 507.3374, 364.1141, 315.8048, 230.8547	unknown
136	16.33	-/230.2471[b]	-1.091	-0.5	$C_{14}H_{28}O$	-	230.2476, 212.2371, 166.9297, 132.0399, 120.9649, 105.9431	unknown
137	16.33	-/362.3250[b]	-1.698	-0.5	$C_{20}H_{40}O_4$	-	362.3259, 344.3150, 308.9617, 256.2631, 238.2510, 212.2372, 190.1427	unknown
138	16.39	-/406.3511[b]	-1.821	-0.5	$C_{22}H_{44}O_5$	-	406.3520, 388.3417, 344.3154, 300.2891, 256.2629, 238.2524, 212.2371, 176.1275, 146.1173, 132.1017	unknown
139	16.46	-/318.2993[b]	-1.950	-0.5	$C_{18}H_{36}O_3$	-	318.2997, 300.2895, 256.2631, 212.2361, 180.7247, 161.9287, 132.1017, 105.3631	unknown
140	16.47	-/415.2102[c]	-1.843	9.5	$C_{24}H_{30}O_6$	-	415.2108, 400.1877, 384.1927, 169.1692, 353.1741, 346.1403, 331.1669, 307.1319, 270.0873, 241.0849, 211.0745	unknown
141	16.50	-/244.2630[b]	-1.765	-0.5	$C_{15}H_{30}O$	-	244.2631, 226.2528, 201.9757, 192.0284, 155.9824, 148.9772, 132.9671	unknown

142	16.50	-/272.2577[b]	-1.748	0.5	$C_{16}H_{30}O_2$	-	272.2599, 256,2618, 254.2475, 212.2377, 183.1615, 159.0796, 134.1015	unknown
143	16.52	305.1767/-	2.023	6.5	$C_{18}H_{26}O_4$	305.1765, 287.1661, 263.1670, 249.1499, 235.1341, 209.1181, 199.8505, 185.1179, 174.9549, 160.8417, 137.0967, 135.0805, 125.0961, 109.0653, 106.0413	-	unknown
144	16.54	-/254.2474[b]	-1.421	1.5	$C_{16}H_{28}O$	-	254.2475, 232.9093, 209.8904, 191.0824, 179.0702, 165.0547, 1432.9024	unknown
145	16.54	-/288.2891[b]	-1.165	-0.5	$C_{17}H_{34}O_2$	-	288.2894, 270.2788, 261.1828, 246.1099, 233.1532, 177.0909, 161.0962, 169.0803, 147.0811, 119.0857, 102.0914	unknown
146	16.56	293.1768/-	2.106	5.5	$C_{17}H_{26}O_4$	293.1765, 282.8307, 238.1115, 236.1055, 223.1615, 221.1544, 207.1389, 205.1231, 192.1149, 177.0914, 164.0832, 148.0522, 134.8937, 116.9275, 103.9190	-	unknown
147	16.57	-/293.2105[c]	-2.085	4.5	$C_{18}H_{28}O_3$	-	293.2105, 275.2001, 267.1125, 257.1984, 247.2055, 229.1946, 205.1219, 191.1432, 175.1112, 149.0959, 119.0857, 107.0494, 95.0495	unknown
148	16.61	-/256.2629[b]	-1.683	0.5	$C_{16}H_{30}O$	-	256.2631, 212.2374, 138.6861, 102.0916, 89.0794	unknown
149	16.65	-/274.2732[b]	-1.480	-0.5	$C_{16}H_{32}O_2$	-	274.2737, 256.2636, 242.6721, 233.1533, 212.2367, 203.1426, 173.1318, 159.1167, 147.1165, 133.1010, 119.0853, 106.0865	unknown

150	16.75	-/376.2587[a]	1.181	3.0	$C_{21}H_{37}O_4$	-	376.2589, 358.2480, 302.1855, 292.2013, 275.1748, 270.2785, 218.1286, 209.1281, 191.1177, 171.3057, 148.5505	unknown
151	16.75	-/449.3476[c]	0.489	0.5	$C_{24}H_{48}O_7$	-	449.3475, 393.9016, 376.2583, 361.9195, 297.1382, 223.0155, 195.5945, 149.4564, 133.0649	unknown
152	16.83	-/453.3353[c]	-1.195	8.5	$C_{30}H_{44}O_3$	-	453.3358, 435.3269, 417.3070, 407.3304, 389.3205, 373.2140, 335.2734, 2676.2104, 247.1696, 219.1747, 213.1635, 205.1584, 201.1636, 187.1480, 173.1322	unknown
153	17.01	-/487.3406[c]	-1.131	7.5	$C_{30}H_{46}O_5$	-	487.3412, 469.3301, 441.3351, 405.3152, 387.3037, 317.2102, 299.1996, 271.2055, 253.1945, 217.1584, 199.1476, 189.1634, 175.1478, 145.1010, 119.0856	unknown
154	17.12	-/415.2020[a]	2.192	6.5	$C_{22}H_{32}O_6$	-	415.2100, 338.5041, 133.0647, 119.0856, 107.0856, 91.0546, 79.0546	unknown
155	17.15	-/286.3094[b]	-1.751	-0.5	$C_{18}H_{36}O$	-	286.3099, 230.2475, 212.2371, 192.2424, 149.1344, 129.9109, 116.9727, 85.1014	unknown
156	17.19	-/323.1244[a]	-1.641	8.5	$C_{18}H_{20}O_4$	-	323.1248, 259.8962, 231.9019, 221.0804, 208.8849, 163.0391, 147.0438, 116.9721, 107.0853	unknown
157	17.24	-/313.2396[b]	-2.605	7.0	$C_{21}H_{27}O$	-	313.2392, 295.2280, 277.2169, 248.9754, 219.1167, 201.1129, 183.1022, 171.0120, 165.0917, 139.1116, 127.1118, 97.0646	unknown

158	17.26	-/437.1921[a]	-2.333	9.5	$C_{24}H_{30}O_6$	-	437.1924, 353.1990, 303.1199, 228.9141, 201.1644, 159.1158, 138.0912, 119.0852, 95.0858	unknown
159	17.29	-/302.3044[b]	-1.707	-0.5	$C_{18}H_{36}O_2$	-	302.3048, 284.2941, 262.6777, 240.2681, 197.4854, 184.1185, 131.0491, 119.0859	unknown
160	17.29	-/394.2943[b]	-1.586	4.5	$C_{23}H_{36}O_4$	-	394.2946, 371.2113, 353.2001, 319.0972, 259.0855, 241.0747, 189.1649, 155.0104, 131.0852, 109.1013	unknown
161	17.38	593.2753/-	-2.117	14.5	$C_{34}H_{42}O_9$	593.2744, 515.4741, 442.4623, 413.2101, 382.3220, 351.7036, 325.5880, 315.0495, 277.2176, 241.0119, 223.0020, 152.9950, 137.6979	-	unknown
162	17.40	-/518.3231[c]	-1.206	8.0	$C_{30}H_{45}O_7$	-	518.3232, 500.3132, 464.9085, 394.7274, 258.1089, 184.0732, 163.0152, 124.9998	unknown
163	17.54	-/284.2939[b]	-2.010	0.5	$C_{18}H_{34}O$	-	284.2942, 279.9095, 261.8950, 251.9107, 233.8998, 208.8833, 192.8730, 176.8817, 164.0700, 145.1001, 132.9669	unknown
164	17.62	-/406.3305[b]	-2.167	4.5	$C_{25}H_{40}O_3$	-	406.3307, 392.8503, 378.9544, 213.4917, 201.0157, 167.2832, 133.1010, 113.3641	unknown
165	17.69	-/277.2155[c]	-2.946	4.5	$C_{18}H_{28}O_2$	-	277.2154, 259.2052, 249.1839, 231.1739, 203.1789, 173.1317, 156.9904, 149.0231, 137.0595, 133.1011, 121.1013	unknown

166	17.73	-/425.3403[a]	2.276	4.5	$C_{27}H_{46}O_2$	-	425.3400, 407.3291, 389.3203, 373.2869, 301.2147, 283.2047, 255.2098, 235.1682, 215.1791, 187.1481, 175.1476, 163.1478, 149.1324, 121.1012	unknown
167	17.78	315.2550/-	1.862	1.5	$C_{18}H_{35}O_4$	315.2547, 311.2265, 297.2440, 279.2328, 253.2534, 239.2371, 201.1137, 171.1021, 155.1067, 141.1275, 127.1117	-	unknown
168	17.78	577.2707/-	-2.336	1.5	$C_{23}H_{46}O_{16}$	577.2700, 410.1085, 299.0456, 277.2176, 225.0076, 206.9964, 164.9857, 152.9856, 134.9750, 106.9796	-	unknown
169	17.80	-/337.2729[c]	-2.554	3.5	$C_{21}H_{36}O_3$	-	337.2729, 316.6897, 305.1812, 296.1780, 284.6686, 257.6517, 235.6574, 224.1504, 215.1451, 187.1477, 161.1319, 147.1165, 133.1010	unknown
170	17.80	-/520.3387[c]	-1.125	7.0	$C_{30}H_{47}O_7$	-	520.3389, 502.3258, 326.2957, 196.2920, 184.0731, 166.0258, 146.3309, 124.9998	unknown
171	17.94	-/200.2006[b]	-1.354	0.5	$C_{12}H_{22}O$	-	200.2006, 178.9600, 169.9771, 158.9860, 146.9611, 137.9674, 135.9701, 133.9746, 128.9506, 119.9659	unknown
172	17.98	-/542.3201[a]	-0.664	7.0	$C_{30}H_{47}O_7$	-	542.3210, 483.2476, 439.2211, 337.2733, 294.9461, 188.0086	unknown
173	18.12	-/374.3619[b]	-1.605	-0.5	$C_{22}H_{44}O_3$	-	374.3623, 356.3525, 312.3255, 268.2983, 254.7480, 216.4242, 175.9781, 146.1172	unknown

174	18.14	471.3495/-	2.348	7.5	$C_{30}H_{48}O_4$	471.3491, 453.3375, 411.3268, 340.7722, 224.6396, 173.1400, 142.4072, 122.5817	-	unknown
175	18.19	-/453.3391[a]	2.500	5.5	$C_{28}H_{46}O_3$	-	453.3351, 435,3246, 407.3314, 389.3192, 335.2746, 311.2356, 267.2086, 253.1953, 225.1642, 215.1794	unknown
176	18.28	233.1552/235.1688[c]	0.415	5.5	$C_{15}H_{22}O_2$	233.1548, 217.1238, 205.8617, 199.8510, 143.8651, 134.8938, 115.9196	235.1689, 190.9869, 179.1064, 172.9770, 163.0750, 140.9510	unknown
177	18.29	-/356.3514[b]	-1.561	0.5	$C_{22}H_{42}O_2$	-	356.3517, 338.3407, 312.3250, 293.2107, 218,8681, 135.1164	unknown
178	18.34	469.3341/-	1.954	8.5	$C_{30}H_{46}O_4$	469.3333, 423.3276, 407.2956, 393.2817, 159.4625, 127.3616	-	unknown
179	18.35	431.2218/-	-2.279	13.5	$C_{28}H_{32}O_4$	431.2218, 277.2186, 241.8959, 191.5173, 171.0058, 156.7762, 152.9951, 144.6298, 115.9201	-	unknown
180	18.40	-/409.3457[c]	-1.545	7.5	$C_{29}H_{44}O$	-	409.3459, 391.3353, 366.0673, 217.1952, 206.1623, 203.1791, 187.1480	unknown
181	18.47	-/471.3456[c]	-0.183	7.5	$C_{30}H_{46}O_4$	-	471.3468, 447.3454, 425.3418, 361.5003, 319.6409, 271.2061, 253.1945, 235.1691, 189.1631	unknown
182	18.54	-/581.4370[a]	-1.884	1.5	$C_{32}H_{62}O_7$	-	581.4377, 559.3466, 397.3805, 329.2189, 244.0728, 216.9783, 164.1066	unknown

183	18.58	-/277.2155[c]	-2.261	4.5	$C_{18}H_{28}O_2$	-	277.2156, 259.2049, 235.1694, 195.1375, 185.1327, 137.0962, 136.1166	unknown
184	18.67	579.2864/-	-2.155	0.5	$C_{23}H_{48}O_{16}$	579.2857, 299.0448, 279.2329, 255.2361, 225.0074, 206.9969, 164.9856, 152.9854, 134.9747, 106.9795	-	unknown
185	18.69	-/473.3614[c]	0.092	6.5	$C_{30}H_{48}O_4$	-	473.3626, 437.3397, 427.3564, 409.3457, 391.3312, 357.2793, 331.2617, 301.2150, 269.2255, 255.2107	unknown
186	18.69	-/457.3665[c]	-1.556	6.5	$C_{30}H_{48}O_3$	-	457.3669, 439.3582, 410.2493, 387.8828, 326.9501, 285.2194, 189.1635, 175.1479, 147.1166	unknown
187	18.72	-/522.3542[c]	-1.523	6.0	$C_{30}H_{49}O_7$	-	522.3543, 504.3430, 431.4034, 390.4995, 297.5789, 258.1093, 226.0755, 184.0731	unknown
188	18.79	-/544.3358[a]	-1.598	6.0	$C_{30}H_{49}O_7$	-	544.3362, 485.2631, 456.4117, 339.2885, 176.9920, 146.9814	unknown
189	18.80	-/313.2727[c]	-1.888	1.5	$C_{19}H_{36}O_3$	-	313.2731, 298.0905, 272.6649, 257.2463, 240.0993, 184.0728, 161.0562, 149.5485, 130.5406	unknown
190	18.90	-/339.2886[c]	-1.979	2.5	$C_{21}H_{38}O_3$	-	339.2887, 281.4410, 265.2521, 228.8878, 185.9368, 163.1483, 149.1324	unknown
191	18.91	571.2910/-	-1.166	11.5	$C_{32}H_{44}O_9$	571.2906, 543.8367, 492.7833, 467.0454, 433.9619, 409.2360, 391.2257, 361.7147, 315.0491, 271.8633, 259.0218, 255.2332, 241.0121, 223.0012, 174.6235, 171.0061, 152.9950, 145.0285, 134.9844, 116.9274,	-	unknown

						111.4863		
192	18.97	-/358.3671[b]	-1.803	-0.5	$C_{22}H_{44}O_2$	-	358.3573, 349.8393, 340.3572, 317.2069, 259.0971, 240.7392, 129.2535	unknown
193	19.05	-/402.3934[b]	-1.618	-0.5	$C_{24}H_{48}O_3$	-	402.3935, 384.3839, 363.1117, 340.3568, 322.3463, 309.2776, 297.0648, 271.0839, 253.0742, 197.0116, 122.0963	unknown
194	19.20	-/437.3405[c]	0.967	8.5	$C_{30}H_{44}O_2$	-	437.3418, 394.2897, 391.3356, 287.2010, 261.1852, 243.2105, 227.1795, 215.1789, 203.1794, 189.1634, 175.1479, 147.1167	unknown
195	19.39	-/427.3559[c]	-0.929	6.5	$C_{29}H_{46}O_2$	-	427.3567, 409.3466, 357.2777, 331.2619, 259.2063, 249.1903, 219.1738, 205.1586, 189.1637, 177.1639, 159.1165	unknown
196	19.41	617.3864/-	1.194	13.5	$C_{39}H_{54}O_6$	617.3855,491.1622,453.3399,401.9221,337.2760,221.9551,196.8956,163.0396,145.0287,121.0286,117.0336	-	unknown
197	19.43	-/337.2729[c]	-1.398	3.5	$C_{21}H_{36}O_3$	-	337.2733, 320.0912, 263.2366, 245.2259, 233.8978, 175.1481, 163.1475, 147.1169, 133.1008	unknown

198	19.55	-/324.2891[b]	-0.666	2.5	$C_{20}H_{34}O_2$	-	324.2895, 306.2784, 284.2952, 261.8951, 245.2259, 210.8846, 189.1631, 175.1481, 161.1328, 135.1168	unknown
199	19.59	-/401.1951[c]	-0.461	9.5	$C_{23}H_{28}O_6$	-	401.1957, 386.1717, 371.1844, 340.1664, 331.1173, 316.0956, 269.0804, 242.0932, 211.0753, 199.0753, 121.1013	unknown
200	19.71	-/501.3902[a]	-1.977	3.5	$C_{30}H_{54}O_4$	-	501.3904, 461.3001, 371.3143, 335.2984, 308.9404, 250.0201, 189.1637, 159.1165	unknown
201	19.74	-/541.3334[a]	-2.114	2.5	$C_{27}H_{50}O_9$	-	541.3336, 528.5671, 419.5198, 311.2939, 284.2924, 256.2633, 147.0437	unknown
202	19.80	511.3444/513.3565[c]	1.960	9.5	$C_{32}H_{48}O_5$	511.3439, 495.3484, 491.4787, 465.3333, 453.3367, 435.2896, 405.3171, 389.2863, 375.2670, 331.2282, 292.7905, 238.4696, 163.2642	513.3568, 496.2130, 67.3527, 435.3263, 407.3277, 390.3221, 317.2108, 271.2052, 235.1688	unknown
203	19.94	-/277.2157[c]	-2.152	4.5	$C_{18}H_{28}O_2$	-	277.2156, 259.2053, 235.1694, 219.9569, 210.0453, 173.1325, 149.0232, 145.1008, 121.1012	unknown
204	19.94	-/546.3517[a]	0.037	5.0	$C_{30}H_{51}O_7$	-	546.3527, 523.5080, 487.2786, 341.3049, 248.5795, 188.0089	unknown
205	20.00	441.3391/-	1.975	7.5	$C_{29}H_{46}O_3$	441.3383, 423.3274, 407.2955, 363.2915, 289.2188, 273.1861, 249.1866, 205.1244, 191.1438, 175.1116, 158.8460, 137.0961, 111.0806	-	unknown
206	20.02	-/226.1797[b]	-1.395	2.5	$C_{13}H_{20}O_2$	-	226.1798, 208.1682, 184.9854, 167.1428, 132.9667, 116.9720, 95.0857	unknown

207	20.02	-/437.3405[c]	-0.794	8.5	$C_{30}H_{44}O_2$	-	437.3411, 391.3353, 285.1850, 201.1634, 145.1011, 119.0856	unknown
208	20.06	-/247.1688[c]	-0.229	5.5	$C_{16}H_{22}O_2$	-	247.1692, 229.1584, 201.1635, 177.0909, 145.1009, 133.1011, 119.0856, 105.0701, 93.0701	unknown
209	20.06	-/280.2628[b]	-1.860	2.5	$C_{18}H_{30}O$	-	280.2630, 263.2368, 245.2260, 219.0567, 161.1327, 149.0231, 109.1013	unknown
210	20.10	-/277.2158[c]	-2.621	4.5	$C_{18}H_{28}O_2$	-	277.2155, 235.1689, 219.0570, 201.0455, 163.1480, 149.0232, 107.0856	unknown
211	20.10	-/550.3859[c]	-0.755	6.0	$C_{32}H_{53}O_7$	-	550.3860, 532.3766, 239.2364, 184.0732	unknown
212	20.14	-/456.4402[b]	-1.777	0.5	$C_{28}H_{54}O_3$	-	456.4403, 437.3394, 283.2631, 255.2315, 189.1634	unknown
213	20.22	-/409.3456[c]	-1.619	7.5	$C_{29}H_{44}O$	-	409.3458, 381.3498, 219.1738, 203.1791, 177.1635, 121.1012	unknown
214	20.26	-/558.4357[b]	-0.698	2.5	$C_{31}H_{56}O_7$	-	558.4360, 447.3471, 256.2631, 237.0974, 171.0135	unknown
215	20.30	-/300.2891[b]	0.180	0.5	$C_{18}H_{34}O_2$	-	300.2898, 239.2367, 204.0649, 123.1171	unknown
216	20.30	-/337.2730[c]	-0.746	3.5	$C_{21}H_{36}O_3$	-	337.2730, 299.0612, 283.0306, 224.1865, 121.1011, 109.1011	unknown
217	20.40	483.2747/-	-2.156	10.5	$C_{29}H_{40}O_6$	483.2742, 440.2310, 418.5778, 255.2333, 245.0436, 227.0327, 152.9951, 142.0308, 130.8746, 171.0056	-	unknown
218	20.42	-/605.4378[a]	-0.090	3.5	$C_{34}H_{62}O_7$	-	605.4382, 256.2628	unknown
219	20.46	-/411.3614[c]	-0.930	6.5	$C_{29}H_{46}O$	-	411.3618, 393.3514, 247.2417, 231.2103,	unknown

							203.1792, 163.1479	
220	20.46	-/439.3577[c]	1.395	7.5	$C_{30}H_{46}O_2$	-	439.3577, 393.3505, 271.2384, 249.1844, 203.1793, 119.0856	unknown
221	20.53	-/365.2682[c]	2.119	4.5	$C_{22}H_{36}O_4$	-	365.2694, 306.2796, 263.2002, 247.7993, 151.0751, 109.0650	unknown
222	20.63	271.2289/-	2.403	1.5	$C_{16}H_{32}O_3$	271.2285, 269.2121, 253.2179, 227.2296, 225.2224, 223.2096, 197.1908, 197,1908, 189.2073, 167.9001, 156.5185, 136.8915, 134.8937, 116.9275	-	unknown
223	20.65	-/528.4980[b]	-1.222	0.5	$C_{32}H_{62}O_4$	-	528.4980, 466.4599, 318.2987, 300.2892, 255.2315, 212.2367, 246.1174	unknown
224	20.77	-/572.5236[b]	2.217	0.5	$C_{34}H_{56}O_5$	-	572.5261, 510.4872, 344.3149, 300.2892, 256.2630, 212.2373, 132.1019	unknown
225	20.78	-/383.2762[a]	-1.684	0.5	$C_{20}H_{40}O_5$	-	383.2762, 364.2848, 303.3470, 191.1785, 110.0603	unknown
226	20.79	581.3024/-	-1.563	-0.5	$C_{23}H_{50}O_{16}$	581.3017, 517.2545, 464.4983, 455.3527, 380.2758, 351.7962, 299.0434, 281.2501, 250.1798, 232.4672, 225.0073, 206.9966, 188.9862, 164.9855, 148.9903, 134.9748, 122.9745, 134.9748, 106.9795, 101.0234	-	unknown
227	20.85	-/637.3037[a]	-0.364	2.5	$C_{27}H_{50}O_{15}$	-	637.3040, 581.2418, 525.1800, 469.1175, 393.0853, 337.0229, 260.9921, 175.0150, 147.1165	unknown
228	20.93	-/313.2733[c]	-1.186	1.5	$C_{19}H_{36}O_3$	-	313.2733, 270.2782, 158.1535, 109.1013	unknown

229	20.94	-/240.1956[b]	-1.813	2.5	$C_{14}H_{22}O_2$	-	240.1954, 199.9757, 181.1584, 135.0802	unknown
230	21.01	-/256.2631[b]	-1.332	0.5	$C_{16}H_{30}O$	-	256.2632, 199.1489, 170.1077, 140.0680, 102.0915	unknown
231	21.02	-/607.4536[a]	0.172	2.5	$C_{34}H_{64}O_7$	-	607.4545, 563.3108, 503.2417, 405.2098, 209.0780	unknown
232	21.10	-/600.5547[b]	-1.417	0.5	$C_{36}H_{70}O_5$	-	600.5553, 494.4921, 364.0432, 344.3156, 283.2627, 256.2626	unknown
233	21.17	-/282.2784[b]	-1.705	1.5	$C_{18}H_{32}O$	-	282.2787, 247.2416, 191.1781, 149.1321, 114.0914	unknown
234	21.25	-/663.4522[b]	0.578	3.0	$C_{34}H_{61}O_{11}$	-	663.4548, 607.3903, 551.3275, 383.1401, 327.0774, 251.0463, 147.1166	unknown
235	21.29	-/568.5652[b]	-0.812	0.5	$C_{36}H_{70}O_3$	-	568.5659, 543.3419, 447.3529, 339.3262, 311.2938, 256.2630, 135.1167	unknown
236	21.32	-/512.5029[b]	-1.661	0.5	$C_{32}H_{62}O_3$	-	512.5029, 311.2927, 283.2625, 228.2319, 149.0592	unknown
237	21.33	-/609.2687[c]	-0.925	14.5	$C_{34}H_{40}O_{10}$	-	609.2689, 549.2478, 475.2086, 405.2086, 285.0104	unknown
238	21.48	-/624.6278[b]	-1.684	0.5	$C_{40}H_{78}O_3$	-	624.6279, 584.6228, 447.3474, 340.3571, 265.2516, 127.0388	unknown
239	21.66	-/628.5861[b]	-1.051	0.5	$C_{38}H_{74}O_5$	-	628.5868, 566.5511, 344.3158, 311.2940, 238.2529	unknown
240	21.70	-/328.3203[b]	0.317	0.5	$C_{20}H_{38}O_2$	-	328.3211, 311.2923, 260.2290, 123.1177	unknown
241	21.71	-/556.5291[b]	-1.143	0.5	$C_{34}H_{66}O_4$	-	556.5293, 538.5180, 301.2923, 283.2626, 256.2631, 146.1173,	unknown

242	21.74	-/338.3409[b]	-1.807	1.5	$C_{22}H_{40}O$	-	338.3411, 321.3149, 226.2160, 163.1478, 121.1012	unknown
243	21.78	-/512.5030[b]	-0.822	0.5	$C_{32}H_{62}O_3$	-	512.5033, 447.3436, 339.2880, 283.2627, 228.2321, 121.1011	unknown
244	21.78	-/226.1797[b]	-1.395	2.5	$C_{13}H_{20}O_2$	-	226.1798, 208.1693, 167.1429, 149.1325, 125.0959, 111.0806	unknown
245	21.81	-/540.5341[b]	-1.224	0.5	$C_{34}H_{66}O_3$	-	540.5344, 447.3436, 256.2631, 212.2367, 121.1008	unknown
246	21.85	-/427.3927[c]	-0.591	5.5	$C_{30}H_{50}O$	-	427.3932, 385.8102, 247.2413, 217.1950, 191.1791, 149.1323, 121.1012	unknown
247	21.85	-/484.4141[b]	-1.107	6.5	$C_{32}H_{50}O_2$	-	484.4144, 426.3721, 276.2316, 191.1792, 135.1167, 110.0966	unknown
248	21.87	497.3654/-	2.547	8.5	$C_{32}H_{50}O_4$	497.3649, 476.5606, 437.3426, 416.2332, 279.8585, 260.0638, 198.9147, 144.8913	-	unknown
249	21.89	-/284.2943[b]	-1.060	0.5	$C_{18}H_{34}O$	-	284.2945,222.0130,119.0856	unknown
250	21.92	-/270.2785[b]	-1.115	0.5	$C_{17}H_{32}O$	-	270.2788, 228.2321, 185.6654, 133.1015	unknown
251	22.00	-/491.3722[c]	-1.793	5.5	$C_{30}H_{50}O_5$	-	491.3722, 447.3457, 339.2900, 263.2351, 173.1172, 109.1014	unknown
252	22.03	-/340.2841[b]	-1.471	2.5	$C_{20}H_{34}O_3$	-	340.2841, 322.2730, 198.1486, 152.1429, 109.1014	unknown
253	22.04	-/283.2627[c]	-2.284	1.5	$C_{18}H_{34}O_2$	-	283.2625, 259.0950, 165.1279, 109.1014	unknown

254	22.18	-/310.3097[b]	-1.938	1.5	$C_{20}H_{36}O$	-	310.3098, 293.2828, 149.1324, 107.0860	unknown
255	22.25	-/540.5338[b]	-1.335	0.5	$C_{34}H_{66}O_3$	-	540.5343, 339.2899, 256.2631, 212.2369, 109.1014	unknown
256	22.26	-/584.5604[b]	-1.396	0.5	$C_{36}H_{70}O_4$	-	584.5604, 447.3438, 311.2939, 256.2631, 146.1173	unknown
257	22.50	-/621.3096[a]	-1.106	2.5	$C_{27}H_{50}O_{14}$	-	621.3086, 509.1847, 453.1229, 335.1765, 279.1143, 223.0516, 147.1166	unknown
258	22.81	-/240.1954[b]	-1.065	2.5	$C_{14}H_{22}O_2$	-	240.1954, 222.1851, 199.1228, 181.1584, 148.9766, 111.0807	unknown
259	23.03	383.3543/-	2.273	1.5	$C_{24}H_{48}O_3$	383.3539, 371.1904, 353.1812, 337.3483, 309.3172, 294.6829, 255.2328, 189.4311, 141.4749, 125.9544, 116.9274	-	unknown
260	23.05	-/410.3168[a]	1.324	5.0	$C_{26}H_{43}O_2$	-	410.3161, 341.2460, 217.1219, 203.1064, 151.0753	unknown
261	23.06	313.2395/-	1.938	2.5	$C_{18}H_{34}O_4$	313.2390, 297.2440, 293.2116, 281.2493, 269.2484, 253.2529, 239.1998, 222.0588, 211.1709, 197.1913, 183.1745, 171.1017, 155.1432, 141.1272, 127.1117, 107.1887	-	unknown
262	23.08	-/312.3254[b]	0.059	0.5	$C_{20}H_{38}O$	-	312.3261, 270.2797, 175.1503, 109.1013	unknown
263	23.16	-/487.2963[b]	2.402	9.0	$C_{28}H_{37}O_6$	-	487.2940, 431.2343, 375.1716, 319.1091, 263.0465, 183.0805	unknown
264	23.27	-/240.1951[b]	-1.272	2.5	$C_{14}H_{22}O_2$	-	240.1955, 222.1849, 199.1224, 181.1585, 152.1069, 111.0805	unknown
265	23.27	-/284.2939[b]	-1.588	0.5	$C_{18}H_{34}O$	-	284.2943, 239.9966, 201.9756, 102.0916	unknown

266	23.49	-/338.3409[b]	-1.630	1.5	$C_{22}H_{40}O$	-	338.3412, 321.3143, 303.3038, 238.1792, 184.1691, 149.1323, 109.1013	unknown
267	23.61	-/226.1798[b]	-1.130	2.5	$C_{13}H_{20}O_2$	-	226.1799, 208.1692, 184.1696, 167.1429, 149.1320, 125.0966, 111.0806	unknown
268	23.85	-/423.2504[a]	1.510	7.5	$C_{25}H_{36}O_4$	-	423.2512, 382.2260, 359.2089, 341.1978, 229.0693, 212.0216, 174.9899, 116.9722	unknown
269	23.98	-/442.3668[b]	-1.257	6.5	$C_{29}H_{44}O_2$	-	442.3674, 384.3243, 316.2624, 194.1539, 133.1012, 108.0810	unknown
270	24.26	311.2967/-	1.980	1.5	$C_{20}H_{40}O_2$	311.2962, 268.0034, 227.0572, 211.5693, 185.5414, 161.0246, 123.8373, 112.3557	-	unknown

[a]: precursor ions referring to $[M+Na]^+$;

[b]: precursor ions referring to $[M+NH_4]^+$;

[c]: precursor ions referring to $[M+H]^+$;

-: not detected or not available.

Table S4 Information of the LLF, EH, and EZP samples analyzed in this work.

Drug	Origin	Manufacturer	Batch Number
**LLF (1–6)**	Hunan Province	Guangyuan Ying pharmacy	-
	Zhejiang Province	Beijing Tongrentang Nankai Pharmacy	-
	Sichuan Province	Huafeng Pharmacy	-
	Shandong Province	Shunshi De Pharmacy	-
	Sichuan Province	Zhongjing Tang Pharmacy	-
	-	Runtu Pharmacy	-
**EH (1–6)**	Henan Province-1	-	-
	Henan Province-2	-	-
	Henan Province-3	-	-
	Hebei Province-1	-	-
	Hebei Province-2	-	-
	Hebei Province-3	-	-
**EZP (1–6)**	-	Tianjin Zhongxin Pharmaceutical Group Co., Ltd.	180001
	-	Tianjin Zhongxin Pharmaceutical Group Co., Ltd.	180001
	-	Jiangxi Renfeng Pharmaceutical Co., Ltd.	170709
	-	Jiangxi Yaodu Zhangshu Pharmaceutical Co., Ltd.	170502
	-	Jiangxi Renfeng Pharmaceutical Co., Ltd.	170306
	-	Jiangxi Renfeng Pharmaceutical Co., Ltd.	170506
